# Genetic Insight into Yield-Associated Traits of Wheat Grown in Multiple Rain-Fed Environments

**DOI:** 10.1371/journal.pone.0031249

**Published:** 2012-02-17

**Authors:** Xianshan Wu, Xiaoping Chang, Ruilian Jing

**Affiliations:** National Key Facility for Crop Gene Resources and Genetic Improvement, Institute of Crop Science, Chinese Academy of Agricultural Sciences, Beijing, China; Cinvestav, Mexico

## Abstract

**Background:**

Grain yield is a key economic driver of successful wheat production. Due to its complex nature, little is known regarding its genetic control. The goal of this study was to identify important quantitative trait loci (QTL) directly and indirectly affecting grain yield using doubled haploid lines derived from a cross between Hanxuan 10 and Lumai 14.

**Methodology/Principal Findings:**

Ten yield-associated traits, including yield per plant (YP), number of spikes per plant (NSP), number of grains per spike (NGS), one-thousand grain weight (TGW), total number of spikelets per spike (TNSS), number of sterile spikelets per spike (NSSS), proportion of fertile spikelets per spike (PFSS), spike length (SL), density of spikelets per spike (DSS) and plant height (PH), were assessed across 14 (for YP) to 23 (for TGW) year × location × water regime environments in China. Then, the genetic effects were partitioned into additive main effects (*a*), epistatic main effects (*aa*) and their environment interaction effects (*ae* and *aae*) by using composite interval mapping in a mixed linear model.

**Conclusions/Significance:**

Twelve (YP) to 33 (PH) QTLs were identified on all 21 chromosomes except 6D. QTLs were more frequently observed on chromosomes 1B, 2B, 2D, 5A and 6B, and were concentrated in a few regions on individual chromosomes, exemplified by three striking yield-related QTL clusters on chromosomes 2B, 1B and 4B that explained the correlations between YP and other traits. The additive main-effect QTLs contributed more phenotypic variation than the epistasis and environmental interaction. Consistent with agronomic analyses, a group of progeny derived by selecting TGW and NGS, with higher grain yield, had an increased frequency of QTL for high YP, NGS, TGW, TNSS, PFSS, SL, PH and fewer NSSS, when compared to low yielding progeny. This indicated that it is feasible by marker-assisted selection to facilitate wheat production.

## Introduction

Wheat (*Triticum aestivum* L.) is one of the most important crops in the world. Grain yield is the most important economic trait in wheat improvement. Being the final product of many processes, it is directly and multilaterally determined by yield-component traits, such as number of spikes per plant (NSP), number of grains per spike (NGS), one-thousand grain weight (TGW), and indirectly affected by other yield-related traits, e.g. plant architecture. Yield and yield associated traits are complex quantitative traits controlled by multiple genes and are highly influenced by environmental conditions [1]. Some yield associated traits are less environmentally sensitive and have higher heritabilities than grain yield [2]. Therefore, it is useful to examine yield associated traits when evaluating yield in order to gather specific information about the genetic control and relationship between yield and related traits crucial for sustained wheat improvement.

Quantitative trait locus (QTL) analysis has provided an effective way to dissect complicated quantitative traits into component loci and to study their individual effects on a specific trait [3]. In a particular genetic background, QTL analysis allows the identification of QTLs that are environmentally relatively stable, thereby providing the breeder with targets for marker assisted variety improvement. QTLs for yield associated traits were previously reported in wheat [4]–[18]. Grain yield and yield associated traits were located on all chromosomes. However, the genetic basis for most of these traits is not well understood, particularly, epistatic QTL × QTL and QTL × environment interactions. A dissection of these interactions is needed to better understand the genetic control of these traits [19].

Drought is one of the most severe constraints to wheat production. Owing to increasing water shortages and uneven distribution of rainfall, it has become an increasingly important problem [20], [21]. Additionally, under normal water conditions, grain yield is affected greatly by environment [22]. Thus, it is meaningful to study the genetic control of yield-associated traits under a range of rain-fed and normal irrigation conditions.

Based on the mixed linear model [23] which partitions genetic effects into additive main effects (*a*), epistatic main effects (*aa*) and their environment interaction effects (*QE*, including *ae* and *aae*), the purpose of this study was to utilize the Hanxuan 10× Lumai 14 doubled haploid population to examine the genetic control of yield and yield-associated traits under different water-related conditions.

## Results

### Phenotypic distribution of yield-associated traits

In the majority of environments Hanxuan 10 was significantly taller (PH 77.00–132.75 cm) and had more spikes (NSP 5.01–11.27), shorter spikes (SL 5.77–8.98 cm), denser spikelets (DSS 1.93–2.43), and fewer grains (NGS 23.94–38.65) than Lumai 14 (Table 1, File S1). Mean DH line values in the majority of environments for NSP, SL, DSS and NGS, and in all environments for PH, were between the parents. There was segregation for all ten traits in the DH population with the coefficients of variation (CV) ranging from 4.0 to 48.0%. All traits except TNSS (CV 5.0%–7.0%) were highly variable; NSSS was the most variable with CVs of 20.0%–48.0%. As DH progeny distribution is wider than the distribution of parental values, transgressive segregation occurred for all traits.

**Table 1:** Phenotypic distributions of yield-associated traits in the wheat parents and DH lines across different environments.

Traits^1^	Parents		DH lines			
	Hanxuan 10	Lumai 14	Mean	Coefficient of variation	Minimum	Maximum
YP (g)	4.46–17.56	3.11–14.13	3.59–14.45	0.16–0.31	1.16–8.49	5.46–24.00
NSP	5.01–11.27	2.82–7.60	4.40–10.68	0.11–0.23	2.20–6.90	6.80–19.30
NGS	23.94–38.65	30.18–43.60	25.04–36.13	0.10–0.22	14.14–26.96	36.38–75.14
TGW (g)	28.44–46.56	26.70–55.01	27.04–45.62	0.10–0.20	13.38–30.66	36.70–74.08
TNSS	15.08–17.55	14.35–17.87	14.05–17.24	0.05–0.07	11.97–14.82	16.42–20.15
NSSS	1.40–4.65	0.94–4.04	1.72–4.48	0.20–0.48	0.38–2.66	4.88–10.28
PFSS	0.69–0.92	0.72–0.94	0.68–0.90	0.04–0.11	0.23–0.72	0.81–0.98
SL (cm)	5.77–8.98	6.38–9.33	5.78–8.69	0.10–0.13	4.32–6.46	7.68–11.52
DSS	1.93–2.43	1.88–2.25	1.98–2.45	0.10–0.15	1.49–1.91	2.67–3.35
PH (cm)	77.00–132.75	56.25–84.50	61.04–106.44	0.13–0.22	40.00–66.20	81.00–141.00

1 YP (g): Yield per plant; NSP: Number of spikes per plant; NGS: Number of grains per spike; TGW (g): 1000-grain weight; TNSS: Total number of spikelets per spike; NSSS: Number of sterile spikelets per spike; PFSS: Proportion of fertile spikelets per spike; SL (cm): Spike length; DSS: Density of spikelets per spike; PH (cm): Plant height.

### Correlations of yield-associated traits

For most environments, YP was significantly correlated with all traits, for example, positive correlations were observed between YP and NSP, NGS, TGW, TNSS, PFSS, SL, and PH (0.16^*^–0.52^****^), whereas there were negative correlations of YP with NSSS (−0.22^**^) and DSS (−0.19^*^) (Table 2). A positive correlation occurred between NGS and PFSS, TGW and PH, and TNSS and NSSS. Weak positive correlations also occurred between NSP and NSSS, between TNSS and DSS, and among NGS, TNSS and SL. The strongest correlation was observed between PFSS and NSSS with a negative correlation coefficient r = −0.98^****^. The negative correlation between SL and DSS was also highly significant (r = −0.85^****^). There were also significant negative correlations among NSP, NGS and TGW, between NSP and PFSS and PH, between NGS and NSSS, and between TGW and DSS, TNSS and PFSS.

**Table 2:** Pearson's correlation coefficients between yield-associated traits in wheat DH lines grown in the different environments.

Traits^1^	YP (g)	NSP	NGS	TGW (g)	TNSS	NSSS	PFSS	SL (cm)	DSS
NSP	0.16^*^ 2								
	−0.03–0.76^****^								
NGS	0.39^****^	−0.31^****^							
	0.16–0.55^****^	−0.37^****^–0.04							
TGW (g)	0.48^****^	−0.32^****^	−0.30^***^						
	0.16^*^–0.63^****^	−0.46^****^–−0.03	−0.41^****^–0.16						
TNSS	0.21^**^	0.06	0.33^****^	−0.13					
	0.01–0.45^****^	−0.15–0.33^****^	0.07–0.55^****^	−0.23^***^–0.13					
NSSS	−0.22^**^	0.29^***^	−0.49^****^	0.03	0.45^****^				
	−0.51^****^–0.09	0.07–0.37^****^	−0.72^****^–−0.36^****^	−0.26^***^–0.29^***^	0–0.52^****^				
PFSS	0.27^***^	−0.32^****^	0.60^****^	−0.06	−0.29^***^	−0.98^****^			
	−0.05–0.54^****^	−0.38^****^–−0.02	0.43^****^–0.83^****^	−0.28^***^–0.24^***^	−0.36^****^–0.17^*^	−0.99^****^–−0.96^****^			
SL (cm)	0.29^***^	−0.04	0.29^***^	0.07	0.27^***^	0.06	−0.01		
	0.16–0.48^****^	−0.29^***^–0.19^*^	0.16–0.44^****^	−0.27^***^–0.29^***^	0.19^*^–0.44^****^	−0.20^*^–0.12	−0.09–0.26^***^		
DSS	−0.19^*^	0.05	−0.09	−0.16^*^	0.25^***^	0.15	−0.12	−0.85^****^	
	−0.26^***^–−0.04	−0.09–0.23^***^	−0.26^***^–0.04	−0.44^****^–0.24^***^	0.08–0.35^****^	0.06–0.39^****^	−0.38^****^–−0.02	−0.89^****^–−0.81^****^	
PH (cm)	0.52^****^	−0.18^*^	0.05	0.62^****^	0.15	−0.05	0.09	−0.03	0.09
	0.04–0.56^****^	−0.25^***^–0.29^***^	−0.10–0.35^****^	0.12–0.72^****^	0.07–0.26^***^	−0.24^***^–0.17^*^	−0.16^*^–0.29^***^	−0.26^***^–0.14	−0.03–0.33^****^

1 YP (g): Yield per plant; NSP: Number of spikes per plant; NGS: Number of grains per spike; TGW (g): 1000-grain weight; TNSS: Total number of spikelets per spike; NSSS: Number of sterile spikelets per spike; PFSS: Proportion of fertile spikelets per spike; SL (cm): Spike length; DSS: Density of spikelets per spike; PH (cm): Plant height;

2 *, **, ***, ****, significant at *P*<0.05, *P*<0.01, *P*<0.005 and *P*<0.0001, respectively; Correlation coefficients between the averaged yield-associated traits are shown on top; Of each correlation pair, the first and the second values are the minimum and maximum correlation coefficient values among 14 (for YP) to 23 (for TGW) environments tested, respectively.

### Analysis of variance (ANOVA) for yield-associated traits

The ANOVA based on the general linear model for genotypes and year × location × water regime environments showed significant differences between genotypes and between environments for all ten traits (Table 3). The F-values for genotypes were 6.22 (*P*<0.0001) (YP) to 123.49 (*P*<0.0001) (PH) and for environments 245.66 (*P*<0.0001) (DSS) to 910.44 (*P*<0.0001) (TNSS). When ANOVA analysis for all traits was performed to determine the significances of differences between genotypes, between year × location (YL) combinations, between water regimes, and between two-factor combinations of genotypes, YL combinations and water regimes, it was clear that water regimes had a large effect on PH and NSP, but did not significantly affect NSSS and DSS. YL had large effects on YP, NGS, TGW, NSSS, PFSS, SL and DSS. The role of water regimes on TNSS was in line with YL (Table 4). The estimated heritabilities varied between 27.2% (YP) and 86.9% (DSS).

**Table 3:** F values of ANOVA for genotypes and environments in wheat DH lines.

Traits^1^	Year × location × water combination	Genotype	*h_2_* (%)
YP (g)	860.91^****^	6.22^****^	27.2
NSP	404.86^****^	8.28^****^	31.3
NGS	501.38^****^	17.07^****^	51.7
TGW (g)	384.02^****^	39.88^****^	62.8
TNSS	910.44^****^	72.15^****^	79.8
NSSS	273.07^****^	23.07^****^	55.1
PFSS	379.77^****^	18.77^****^	49.7
SL (cm)	636.13^****^	99.44^****^	84.5
DSS	245.66^****^	120.67^****^	86.9
PH (cm)	609.12^****^	123.49^****^	84.8

1 YP (g): Yield per plant; NSP: Number of spikes per plant; NGS: Number of grains per spike; TGW (g): 1000-grain weight; TNSS: Total number of spikelets per spike; NSSS: Number of sterile spikelets per spike; PFSS: Proportion of fertile spikelets per spike; SL (cm): Spike length; DSS: Density of spikelets per spike; PH (cm): Plant height.

**Table 4:** F values of ANOVA-GLM for DH line genotypes and environments as well as their interactions.

Sources	YP (g)^2^	NSP	NGS	TGW (g)	TNSS	NSSS	PFSS	SL (cm)	DSS	PH (cm)
Genotype	7.7^****^	9.9^****^	22.4^****^	64.6^****^	114.9^****^	30.3^****^	24.8^****^	162.9^****^	200.4^****^	257.9^****^
YL^1^	2144.9^****^	841.5^****^	1198.6^****^	1134.6^****^	2543.9^****^	713.2^****^	1014.8^****^	1867.4^****^	746.3^****^	1852.9^****^
Water regime	485.3^****^	1047.5^****^	226.3^****^	560.7^****^	2690.4^****^	0.2	34.7^****^	982.2^****^	0.7	6486.7^****^
YL×Genotype	1.5^****^	1.4^****^	1.6^****^	2.1^****^	2.1^****^	1.6^****^	1.7^****^	2.3^****^	2.4^****^	3.1^****^
Water×Genotype	1.4^****^	1.1	1.5^****^	2.5^****^	2.1^****^	1.2^*^	1.2	1.5^***^	1.4^***^	2.7^****^
YL×Water regime	105.0^****^	46.3^****^	101.5^****^	62.1^****^	198.4^****^	48.4^****^	45.4^****^	225.2^****^	121.3^****^	169.4^****^

1 YL: year × location combination;

2 YP (g): Yield per plant; NSP: Number of spikes per plant; NGS: Number of grains per spike; TGW (g): 1000-grain weight; TNSS: Total number of spikelets per spike; NSSS: Number of sterile spikelets per spike; PFSS: Proportion of fertile spikelets per spike; SL (cm): Spike length; DSS: Density of spikelets per spike; PH (cm): Plant height.

### Genomic distribution of QTLs for yield-associated traits

A total of 241 QTLs controlling yield-associated traits were detected. The number of QTLs for individual traits ranged from 12 to 33 (Table 5). These were unevenly distributed on all chromosomes except 6D, on which no QTL was identified. They were more frequently observed on chromosomes 1B, 2B, 2D, 5A and 6B (more than 16 QTLs). The highest QTL number (22 or 9.1%) was identified on chromosome 2D, whereas the lowest number (2 or 0.8%) was on chromosome 4D. Chromosomes 2B and 7A possessed QTLs associated with all traits, whereas chromosomes 1D (DSS and PH) and 4D (SL and PH) possessed QTLs for only two traits. The number of QTLs on homoeologous groups 1 to 7 ranged from 27 (11.2%) to 48 (19.9%). The QTL frequency was the highest for the B genome with 104 (43.2%), and the lowest in the D genome with 45 (18.7%).

**Table 5:** Distributions of QTLs for yield associated traits in wheat DH lines across chromosomes.

Traits^1^	Numbers of QTLs																					
	Total	1A	1B	1D	2A	2B	2D	3A	3B	3D	4A	4B	4D	5A	5B	5D	6A	6B	6D	7A	7B	7D
YP (g)	12	2			1	1	1		1	1		2			1					1		1
NSP	17	1	2			1	1	1			1	1		1		2	2	1		2	1	
NGS	21	2				1	2	2			1			2			1	4		1	2	3
TGW (g)	32		1			2	2	4	5	1	2	2		4	2	1	1	1		1	3	
TNSS	30	2	5		1	1	3		1		3	2		1	3	1	2	2		1	2	
NSSS	24		3		2	1	2	1				1		1	2	1	4	1		2	1	2
PFSS	16		1		2	1		1			1	2		2	1		1	2		1	1	
SL (cm)	30		2			5	5	1	2		2	3	1	4		1	1			2	1	
DSS	26			3	3	2	2	3	2	1	1			1	1		2	1		2	2	
PH (cm)	33	3	5	2	1	1	4	1	3		1		1	1		1	1	6		1	1	
Total	241	10	19	5	10	16	22	14	14	3	12	13	2	17	10	7	15	18		14	14	6
Homeologous Group		34			48			31			27			34			33			34		
Genome		92	104	45																		

1 YP (g): Yield per plant; NSP: Number of spikes per plant; NGS: Number of grains per spike; TGW (g): 1000-grain weight; TNSS: Total number of spikelets per spike; NSSS: Number of sterile spikelets per spike; PFSS: Proportion of fertile spikelets per spike; SL (cm): Spike length; DSS: Density of spikelets per spike; PH (cm): Plant height.

**Figure 1:**
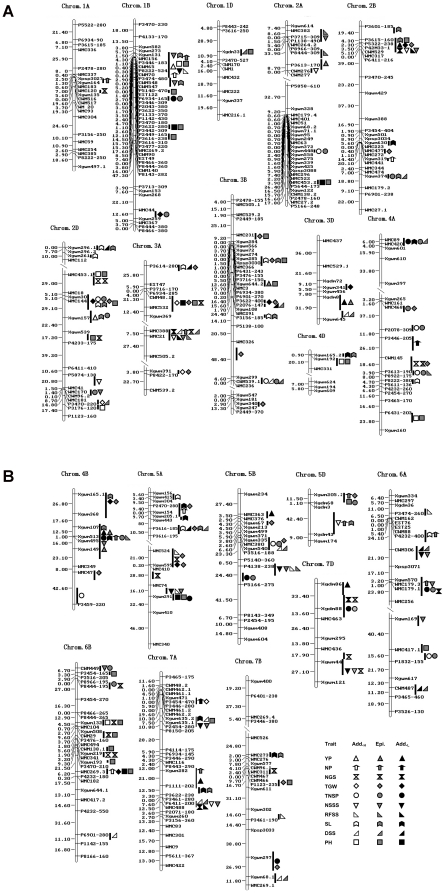
QTL and QTL clusters for yield-associated traits in wheat DH lines. Add_H_, additive QTL contributed by Hanxuan 10; Add_L_, additive QTL contributed by Lumai 14; Epi, QTLs involved in epistatic effects. The bold line indicates the confidence intervals. All additive QTLs are shown; epistatic QTLs are those with large effects (i.e. the phenotypic variation explained (PVE)>the mean PVE of all main effects) and present in QTL clusters. Map distances in centiMorgans (cM) are shown on the left side of each chromosome.

The map positions of QTLs for various yield-associated traits tended to cluster on each chromosome. Many QTLs attributed to correlated traits were clustered in relatively short intervals (10 cM) on chromosomes other than 3D and 6D (Figure 1). QTLs controlling correlated SL and DSS formed QTL clusters on chromosomes 2B, 2D, 3A, 4A, 5A and 7A. QTL clusters controlling PH and TGW were on 2B, 2D, 3A, 3B, 6B and 7B. QTL clusters for NSSS and PFSS were on 1B, 2A, 2B, 3A, 4B, 5A, 5B and 7A. Three QTL clusters associated with grain yield were located on chromosomes 1B, 2B and 4B. The QTL cluster at *Xgwm131-1B* - *Xcwm70*-*1B* was associated with six associated traits, viz. YP, NSP, NSSS, PFSS, SL and PH, and the favorable alleles of all *a* effect QTLs of YP, NSP, NSSS, PFSS and PH were contributed by the female parent Hanxuan 10. In contrast, the favorable alleles of all *a* effect QTLs in the *P3615*-*160-2B* - *Xcwm529-2B* and *Xgwm107-4B* - *Xgwm149-4B* clusters were from the male parent Lumai 14. The 2B group included seven traits (YP, NGS, TGW, PFSS, NSSS, SL and PH) whereas the 4B cluster involved six traits (YP, NSP, TNSS, NSSS, PFSS and SL) (Figure 1).

### Genetic main effects of QTL for yield-associated traits

Thirty three percent (PH) to 66.7% (YP) of QTLs were identified with significant *a* effects, and 50.0% (YP) to 97.0% (PH) of QTLs were involved in significant *aa* effects (Table 6). Of the identified *a* effect QTLs, a significant proportion (22.2% for PFSS - 90.9% for PH) was involved in epistatic interactions with background loci, whereas the majority (51.9% for TNSS - 100.0% for NSP) of loci involved in epistatic interactions did not appear to have significant additive effects (File S2). The *a* effects of Hanxuan 10 alleles increased NSP, SL, DSS, PFSS and PH at 52.6%–66.7% of loci, whereas those of Lumai 14 alleles increased YP, TGW and NGS at 62.5%–75.0% of loci, and the two parent alleles each increased TNSS and NSSS at 50.0% of loci (File S2). Of the *aa* effects, 52.9%–75.0% of epistatic pairs appeared to be recombinant for YP, TGW, NSSS, SL and PH, whereas 53.3%–66.7% of epistatic pairs were parental types for NSP, TNSS, PFSS and DSS. The frequencies of both recombinant and parental types for NGS were about 50.0% (File S2). This suggested that alleles increasing yield-associated traits were inherited from the two parents.

**Table 6:** Numbers, main effects and contributions of QTLs for yield-associated traits in wheat DH lines.

Traits^1^	Numbers of QTLs^2^			Effects (*a*/*aa*)	PVE (*a*/*aa*)(%)
	*a*	*a* and *aa*	*aa*		
YP (g)	6	2	4	0.263/0.183	0.39/0.10
NSP	6		11	0.232/0.174	0.83/0.38
NGS	3	5	13	0.984/0.598	1.46/0.41
TGW (g)	5	8	19	0.942/0.570	1.54/0.46
TNSS	3	13	14	0.206/0.086	1.76/0.26
NSSS	4	9	11	0.136/0.113	1.29/0.46
PFSS	7	2	7	0.009/0.009	1.16/0.52
SL (cm)	11	8	11	0.183/0.138	2.00/0.50
DSS	4	10	12	0.052/0.027	3.68/0.63
PH (cm)	1	10	22	3.445/1.579	2.39/0.57

1 YP (g): Yield per plant; NSP: Number of spikes per plant; NGS: Number of grains per spike; TGW (g): 1000-grain weight; TNSS: Total number of spikelets per spike; NSSS: Number of sterile spikelets per spike; PFSS: Proportion of fertile spikelets per spike; SL (cm): Spike length; DSS: Density of spikelets per spike; PH (cm): Plant height;

2 *a*: additive main effects; *aa*: epistatic main effects; Effects: the mean of absolute additive main effects (*a*)/the mean of absolute epistatic main effects (*aa*); PVE: the mean of phenotypic variations explained by additive main effects (*a*)/the mean of phenotypic variations explained by epistatic main effects (*aa*).

**Figure 2:**
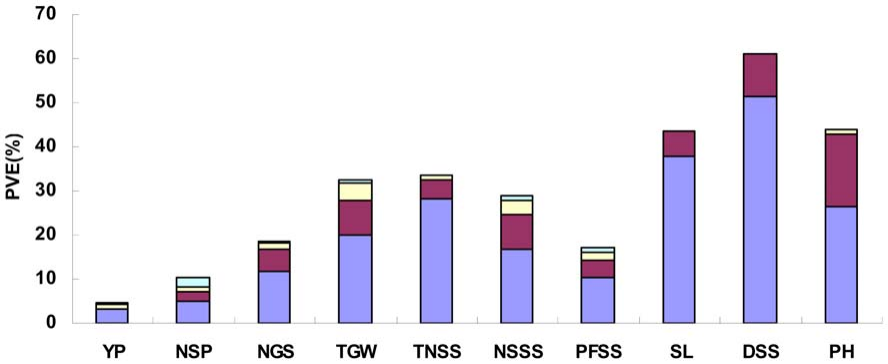
Phenotypic variation explained (PVE) by different genetic components for yield-associated traits in wheat DH lines.

The *a* effect of a single QTL for an individual trait was equal to or more than the *aa* effect of a single epistasis, and the mean phenotypic variation explained due to the *a* effect exceeded that of *aa* effect (Table 6). Overall, the cumulative *a* effects controlling individual traits contributed more than *aa* effects (Figure 2). Thus, additive QTLs were more important than epistatic QTLs for all traits.

### QTL × environment effects for yield-associated traits

For all traits, excluding DSS, 5.3% (SL) - 76.9% (TGW) of additive QTLs were involved in *ae* effects in 1–7 environments across all environments except hd99T (Haidian, Beijing in 1999 under drought stressed treatment (DS)), cp05T (Changping, Beijing in 2005 under DS) and cp06C (Changping, Beijing in 2006 under well-watered treatment (WW)), and for YP, NP, NGS, TGW, NSSS and PFSS, 11.8% (TGW) - 83.3% (NSP) of epistatic pairs were subjected to *aae* effects in one to three of the 11 environments (File S2).

Among additive QTLs involved in *ae* effects, even the absolute value of the *ae* effect of a single QTL for NSP, NGS, TGW, TNSS, SL and PH appeared less than the *a* effect, but for YP, PFSS and NSSS the *ae* effects were greater than the *a* effects (Table 7). *QNSSS.cgb-4B* had only *ae* effects contributed by Hanxuan 10 in environments fy01T (Fenyang, Shanxi in 2001 under DS) and fp99C (Fuping, Shaanxi in 1999 under WW), indicating it was induced by the environment. Thus, the additive effects for YP, PFSS and NSSS were readily affected by environments. In addition, owing to *ae* effects, Hanxuan 10 possessed alleles increasing YP, NSP, NGS, TGW, TNSS, PFSS, SL, and PH, and decreasing NSSS at one to six QTLs in one to three drought stressed environments, suggesting that these QTLs might adapt well to drought conditions (File S2). For example, *QNGS.cgb-2B* conferred positive *ae* effects in fy01T and hd05T (Haidian, Beijing in 2005 under DS), *QTNSS.cgb-2D.1* in ly99T (Luoyang, Henan in 1999 under DS), fp99T (Fuping, Shaanxi in 1999 under DS) and fy01T, *QPH.cgb-6B.6* in fp00T and fy01T, and *QTGW.cgb-6B* in ly99T, fp99T and fy01T. However, some QTLs controlling NSP, NGS, TGW, TNSS, SL and PH with *a* effects contributed far more phenotypic variation than *ae* effects (Table 8). They were less affected by environments and therefore might be beneficial to wheat genetic improvement. In general, the *a* effects (3.1%–38.0%) of all traits contributed more than *ae* effect (0.2%–4.1%) (Figure 2).

**Table 7:** Numbers and environmental interaction effects of QTLs for yield associated traits in wheat DH lines.

Traits^1^	Additive effects				Epistatic effects			
	QTL Numbers		Effects^3^ (*a*/*ae*)	PVE^4^ (*a*/*ae*) (%)	QTL pair numbers		Effects (*aa*/*aae*)	PVE (*aa*/*aae*) (%)
	*a* 2	*ae*			*aa*	*aae*		
YP (g)	8	3	0.243/0.337	0.25/0.28	3	2	0.199/0.330	0.13/0.23
NSP	6	2	0.342/0.210	1.16/0.47	6	5	0.191/0.222	0.45/0.42
NGS	8	4	0.961/0.915	1.50/0.38	12	2	0.800/1.040	0.17/0.24
TGW (g)	13	10	1.037/0.567	1.73/0.41	17	2	0.381/0.568	0.11/0.21
TNSS	16	4	0.208/0.092	1.85/0.29	17	/	/	/
NSSS	12	7	0.094/0.115	0.33/0.46	17	3	0.105/0.110	0.43/0.34
PFSS	9	3	0.006/0.008	0.48/0.60	7	3	0.007/0.011	0.72/0.57
SL (cm)	19	1	0.305/0.078	6.08/0.24	11	/	/	/
DSS	14	/	/	/	15	/	/	/
PH (cm)	11	3	5.2/1.6	3.67/0.36	29	/	/	/

1 YP (g): Yield per plant; NSP: Number of spikes per plant; NGS: Number of grains per spike; TGW (g): 1000-grain weight; TNSS: Total number of spikelets per spike; NSSS: Number of sterile spikelets per spike; PFSS: Proportion of fertile spikelets per spike; SL (cm): Spike length; DSS: Density of spikelets per spike; PH (cm): Plant height;

2 *a*: additive main effects; *aa*: epistatic main effects; *ae*: additive environment interaction effects; *aae*: epistatic environment interaction effects;

3 Effects: the mean of absolute additive main effects (*a*)/the mean of absolute additive environment interaction effects (*ae*);

4 PVE: the mean of phenotypic variations explained by additive main effects (*a*)/the mean of phenotypic variations explained by additive environment interaction effects (*ae*); the same Effects and PVE for *aa*/*aae*.

**Table 8:** Major QTLs in wheat DH lines.

Traits^1^	QTL	Near markers	Additive effects^4^	Parents^5^	PVE (%)	Increases (%)^6^	Total increases (%)^7^
YP	***QYP.cgb-2D*** 2	*Xgwm157*	0.423	Hanxuan 10	1.07	3.74	3.74
NSP	***QNSP.cgb-6B*** 3	*Xwmc269.3*	0.367	Hanxuan 10	1.40	2.56	2.56
NGS	***QNGS.cgb-2B***	*P4233.1*	−0.935	Lumai 14	2.82	4.76	9.95
	***QNGS.cgb-7A***	*Xwmc488*	−1.682	Lumai 14	2.56	5.19	
TGW	***QTGW.cgb-2D.2***	*P3470.3*	1.938	Hanxuan 10	4.90	3.54	7.28
	*QTGW.cgb-3A.4*	*P8422*	−1.376	Lumai 14	2.87	3.73	
TNSS	***QTNSS.cgb-4B.1***	*Xgwm513*	−0.292	Lumai 14	4.77	2.00	4.11
	***QTNSS.cgb-5A***	*Xgwm291*	−0.541	Lumai 14	7.43	2.11	
NSSS	***QNSSS.cgb-5A***	*Xgwm291*	−0.361	Lumai 14	7.18	10.88	17.68
	***QNSSS.cgb-7A.2***	*Xwmc488*	0.250	Hanxuan 10	3.77	6.80	
SL	***QSL.cgb-2D.1***	*Xgwm296.1*	0.173	Hanxuan 10	3.09	4.39	19.89
	*QSL.cgb-2D.2*	*Xwmc112*	0.196	Hanxuan 10	2.73	5.18	
	***QSL.cgb-2D.3***	*Xwmc144*	0.067	Hanxuan 10	3.47	1.57	
	***QSL.cgb-4B.3***	*Xgwm513*	−0.110	Lumai 14	4.02	2.21	
	*QSL.cgb-5A.1*	*Xgwm304*	0.435	Hanxuan 10	2.40	3.09	
	***QSL.cgb-7A.1***	*Xgwm635.1*	−0.305	Lumai 14	6.08	3.45	
DSS	***QDSS.cgb-3B.1***	*P2076*	−0.051	Lumai 14	4.16	2.54	11.16
	*QDSS.cgb-3D*	*Xgwm645*	0.054	Hanxuan 10	5.42	2.22	
	***QDSS.cgb-6A.1***	*Xcwm306*	−0.107	Lumai 14	14.31	3.48	
	***QDSS.cgb-7A.1***	*Xgwm635.1*	0.038	Hanxuan 10	4.41	2.92	
PFSS	***QPFSS.cgb-5A.2***	*Xgwm291*	0.014	Hanxuan 10	2.59	2.55	4.75
	***QPFSS.cgb-7A***	*Xwmc488*	−0.015	Lumai 14	2.10	2.20	
PH	***QPH.cgb-1B.1***	*P3446.1*	5.4	Hanxuan 10	4.68	4.10	20.60
	***QPH.cgb-2D.1***	*Xwmc453.1*	1.9	Hanxuan 10	3.47	5.88	
	***QPH.cgb-4D***	*Xgwm192*	4.3	Hanxuan 10	5.94	6.79	
	***QPH.cgb-6B.6***	*Xwmc269.3*	−6.3	Lumai 14	4.26	3.84	

1 YP (g): Yield per plant; NSP: Number of spikes per plant; NGS: Number of grains per spike; TGW (g): 1000-grain weight; TNSS: Total number of spikelets per spike; NSSS: Number of sterile spikelets per spike; PFSS: Proportion of fertile spikelets per spike; SL (cm): Spike length; DSS: Density of spikelets per spike; PH (cm): Plant height;

2 QTLs in bold were clustered with other QTLs in the same region;

3 Underlined indicates that the QTL has environmental interaction effects;

4 The additive main effects (*a*) of QTL, a positive value indicates that Hanxuan 10 contributes allele to increase the trait, and a negative value means that Lumai 14 provides allele to increase the trait;

5 Parent contributing the allele increasing QTL trait value;

6 Increase relative to the population mean;

7 Total increases in ratio over the population mean.

For epistatic QTLs involved in *aae* effects, generally the averaged absolute value of the *aae* effects detected for epistatic QTL pairs were larger than their *aa* effects, indicating that these epistatic pairs of YP, NSP, NGS, TGW, NSSS and PFSS were highly sensitive to environments (Table 7). Epistatic pairs between *QTGW.cgb-5A.4* and *QTGW.cgb-5B.2* showed positive effects in fy01T, and epistatic pairs between *QNSSS.cgb-4B* and *QNSSS.cgb-6A.1* exhibited negative effects in fy01T. These epistatic effects were beneficial under drought conditions although they contributed very little (File S2). In addition, the total *aa* effects (2.3%–7.9%) accounted for more phenotypic variation than *aae* effects (0.4%–2.1%) for all traits except YP (Figure 2).

### QTL response to selecting high NGS and TGW

Among four categories of progeny lines (high TGW and high NGS group (Hgw_Hgn), high TGW but low NGS group (Hgw_Lgn), low TGW but high NGS group (Lgw_Hgn) and low TGW and low NGS group (Lgw_Lgn)), significant differences occurred for all traits except YP, their F-values were 2.99 (*P*<0.05) - 28.05 (*P*<0.0001) respectively (Table 9). No significant differences observed for YP among four groups indicated that high yield may be achieved by various selection approaches. The comparison between the highest yielding group (Hgw_Hgn) and the lowest yielding group (Lgw_Lgn) suggested that the grain yield and grain weight in these high-yielding lines was associated with a combination of traits, including a heavier TGW and taller plants with a longer SL, a more TNSS, a higher PFSS, a sparser DSS but a fewer NSP and NSSS (Table 9). The two high grain weight groups have a significantly heavier TGW and taller PH than the other two groups, the Hgw_Hgn group has a significantly fewer TNSS than the Lgw_Hgn group. The two high grain number groups have a significantly longer SL, a higher PFSS but lower NSSS than the two low grain number groups. The Hgw_Hgn group has a more TNSS than the Hgw_Lgn group, and the Lgw_Hgn group has a more NGS but a sparser DSS than the Lgw_Lgn group.

**Table 9:** Performances for traits of four 1000 grain weight (gw) - grain number per spike (gn) groups of wheat DH lines from analyses across different environments.

Traits^1^	YP	NSP	NGS	TGW	TNSS	NSSS	PFSS	SL	DSS	PH
F value	1.32	2.99^*^	4.28^**^	28.05^****^	18.51^****^	5.31^***^	5.32^***^	10.68^****^	7.44^***^	25.01^****^
Hgw_Hgn^2^	10.71A^3^	6.98B	33.13B	40.04A	16.98B	2.27 B	0.86A	8.24A	2.11B	98.96A
Hgw_Lgn	9.29A	7.96BA	25.32B	39.84A	16.04A	3.04 A	0.81B	7.49B	2.15B	96.38A
Lgw_Hgn	9.51A	8.19BA	34.75A	28.94B	17.05A	2.39 B	0.86A	8.22A	2.11B	78.32B
Lgw_Lgn	7.83A	9.02A	25.33B	30.18B	16.01A	3.12 A	0.80B	7.09B	2.27A	72.91B
Grain weight^4^	1.66	5.35^*^	0.18	84.86^****^	17.56^****^	0.25	0.16	1.01	3.22	71.80^****^
Grain number	2.30	3.29	31.50^****^	0.11	22.32^****^	16.02^***^	16.18^****^	29.11^****^	12.40^***^	1.30

1 YP (g): Yield per plant; NSP: Number of spikes per plant; NGS: Number of grains per spike; TGW (g): 1000-grain weight; TNSS: Total number of spikelets per spike; NSSS: Number of sterile spikelets per spike; PFSS: Proportion of fertile spikelets per spike; SL (cm): Spike length; DSS: Density of spikelets per spike; PH (cm): Plant height;

2 Hgw_Hgn: high TGW and high NGS group; Hgw_Lgn: high TGW but low NGS group; Lgw_Hgn: low TGW but high NGS group; Lgw_Lgn: low TGW and low NGS group. The sign meanings are consistent in other table;

3 Group means with the same letter are not significantly different;

4 Grain weight indicates grouping based on TGW, namely high TGW (including Hgw_Hgn and Hgw_Lgn) and low TGW (including Lgw_Hgn and Lgw_Lgn); Grain number indicates grouping based on NGS, namely high NGS (including Hgw_Hgn and Lgw_Hgn) and low NGS (including Hgw_Lgn and Lgw_Lgn).

QTL allele frequency at the 34 genomic regions associated with QTL for ten traits was assessed in the 41 progeny lines present in the four groups. The high-yielding progenies were enriched for QTL that resulted in increased YP (one of three QTL from Hanxuan 10), NSP (one QTL), NGS (two of four QTL), TGW (one of six QTL), TNSS (four of six QTL), PFSS (two of four QTL), SL (two of four QTL), DSS (one of three QTL) and PH (one QTL), and reduced NSSS (three QTL from Lumai 14) (Table 10). Of these 25 regions, five varied in frequency between the high and low grain weight groups, resulting in an increased frequency of QTL for higher NSP (one QTL from Hanxuan 10), TGW (one of two QTL), TNSS (one QTL), and DSS (one of two QTL); Seven regions varied between the high and low grain number groups, they were associated with more YP (one of two QTL), NGS (two of three QTL), TNSS (one of two QTL), PFSS (one QTL), SL (one of two QTL), PH (one QTL) and fewer NSSS (one QTL) in the high grain number groups.

**Table 10:** Favorable allele frequencies of the additive main QTLs for yield and yield-related traits in four 1000 grain weight (gw) - grain number per spike (gn) groups of wheat DH lines.

Traits^1^	Chromosomes	QTL marker^2^	Hgw_Hgn	Hgw_Lgn	Lgw_Hgn	Lgw_Lgn	Favourable parents^3^
YP	2D	*Xgwm157*	**1.00** 4	0.54	0.73	0.44	Hanxuan 10
	3D	*Xgdm8*	0.50	**0.92**	0.27	0.22	Lumai 14
YP, TGW	2B	*P5322*	**0.75**	0.54	0.53	0.00	Lumai 14
YP, TNSS, SL	4B	*Xgwm513*	**0.75**	0.38	**0.73**	0.22	Lumai 14
NSP	1B	*Xcwm70*	**0.75**	**0.77**	0.60	0.44	Hanxuan 10
	2B	*Xgwm319*	0.50	0.69	0.47	**1.00**	Hanxuan 10
	6B	*Xwmc269.3*	0.50	0.54	**0.80**	**0.89**	Hanxuan 10
NGS	4A	*Xcwm145*	**1.00**	0.31	**0.80**	0.67	Hanxuan 10
	6B	*Xgwm219*	**1.00**	0.54	**0.87**	**0.78**	Hanxuan 10
NGS, NSSS, PFSS	2B	*P4233.1*	**0.75**	0.46	0.47	0.00	Lumai 14
	3A	*Xwmc21*	**1.00**	0.46	0.67	0.22	Lumai 14
TGW	2D	*P3470.3*	**1.00**	0.62	0.47	0.44	Hanxuan 10
	3A	*Xwmc532*	0.50	**0.77**	0.53	0.44	Hanxuan 10
	3A	*P8422*	**0.75**	0.54	0.33	0.67	Lumai 14
	3D	*Xgwm341*	**0.75**	**0.92**	0.33	0.33	Lumai 14
	4B	*Xgwm368*	0.25	0.46	**0.73**	0.33	Lumai 14
	5A	*Xgwm595*	**0.75**	0.69	0.53	0.56	Lumai 14
TGW, DSS	3B	*P2076*	**0.75**	0.46	0.47	0.56	Lumai 14
TNSS	2A	*Xgwm448*	0.25	**0.77**	0.53	0.44	Hanxuan 10
	4A	*P2078*	**1.00**	0.46	0.53	0.44	Hanxuan 10
	4A	*P3613.2*	**1.00**	0.23	0.67	0.56	Hanxuan 10
	4B	*P3459.1*	0.00	**0.85**	0.67	0.67	Hanxuan 10
	7B	*Xgwm297*	**0.75**	0.46	0.47	0.22	Lumai 14
TNSS, SL	2D	*Xwmc144*	**0.75**	0.38	**0.80**	0.56	Hanxuan 10
TNSS, DSS	3B	*Xcwm539.1*	**1.00**	**0.92**	0.67	0.67	Hanxuan 10
NSSS	5D	*Xgdm43*	**0.75**	0.23	0.33	0.44	Lumai 14
PFSS	5A	*Xwmc524*	**0.75**	0.46	0.33	0.44	Hanxuan 10
	6A	*P3474.2*	**0.75**	0.46	0.60	0.44	Hanxuan 10
SL	3A	*P3614*	**1.00**	0.54	0.53	0.44	Hanxuan 10
	7B	*Xwmc273*	**0.75**	0.69	0.47	0.33	Lumai 14
DSS	3A	*Xwmc21*	0.00	0.54	0.33	**0.78**	Hanxuan 10
	6A	*Xcwm487*	**0.75**	**0.77**	0.60	0.33	Lumai 14
	7A	*Xgwm635.1*	0.50	0.54	**0.73**	0.56	Hanxuan 10
PH	2D	*P3176.1*	0.50	**0.77**	0.60	0.33	Hanxuan 10
	6B	*Xgwm132*	**0.75**	0.69	**0.73**	0.67	Hanxuan 10

1 YP (g): Yield per plant; NSP: Number of spikes per plant; NGS: Number of grains per spike; TGW (g): 1000-grain weight; TNSS: Total number of spikelets per spike; NSSS: Number of sterile spikelets per spike; PFSS: Proportion of fertile spikelets per spike; SL (cm): Spike length; DSS: Density of spikelets per spike; PH (cm): Plant height;

2 The nearest marker of the associated QTL;

3 The parent contributing the favourite allele of QTL;

4 QTL favourite allele frequence in a group, viz, for a special marker, the number of favourite allele divided by the total line number. Approximately the value 0.70 is considered that the favourite allele is rich in the group, which is expressed in bold print.

## Discussion

Grain yield is a major goal for the improvement of wheat in drought-prone areas [21]. Few reports show the genetic basis of yield-associated traits under different water conditions. Maccaferri et al. (2008) investigated the genetic basis of three traits (grain yield, heading date and plant height) in 249 durum wheat recombinant inbred lines in 10 rainfed and 6 irrigated environments [21]. McIntyre et al. (2010) evaluated genomic regions of grain yield and yield-related components in 192 bread wheat recombinant inbred lines under 8 irrigated and rainfed conditions [18]. By association mapping, the genetic basis of grain yield was dissected in a collection of 189 elite durum wheat accessions evaluated in 15 environments highly different for water availability [24]. The present study identified QTL controlling ten yield-associated traits (yield, yield components and other agronomic traits) in a winter wheat DH population under a range of environments (14–23 year × location × water regime combinations) that differed widely in the amounts of available water. In our study, yield was defined as yield per plant as reported in other studies [14], [25]. And the middle five plants in each plot being randomly sampled for analysis was to avoid burdensome measurement, which can represent the plot [25], although the number appeared less in comparison with other studies [26], [27].

Grain yield and yield associated traits are complex quantitative traits controlled by multiple genes and highly affected by environments [1]. An important aspect of this study was the use of the mixed linear model to permit division of genetic effects into additive main effects (*a* effects), additive × additive epistatic main effects (*aa* effects) and their environmental interaction effects (*ae*, *aae*). To date, the majority of QTL studies in wheat have not examined interactions (*ae*, *aa* and *aae*) [2], [4]–[9], [11]–[14], [16], [17], [21], [28]–[30]. QTL analyses not enabling detection of these interactions would lead to biased estimates of main-effect QTLs, particularly for those involved in complex traits like yield. This could result in a lower than predicted genetic gain from marker-assisted selection as well as some difficulties when trying to isolate the QTL [31]. The importance of epistasis and environmental interactions in the genetics of yield traits has been demonstrated in rice and maize [25], [32]–[34]. Results such as those of Zhuang et al. (2002) implied that the detection of QTLs with main effects, as well as the magnitude and directions of the additive effects, might vary depending on their interactions with other loci. Our study also confirmed that all traits evaluated were controlled by *a* effects, *aa* effects and environmental interaction effects (*ae* and/or *aae*), except for density of spikelets per spike (DSS) where no QTL × environment interaction effects (*QE* effects) were detected (Figure 2). We also found that many QTLs with additive effects were involved in interactions with other QTLs that were affected by genetic background (File S2). Although both *a* and *aa* effects contributed to the genetic basis of grain yield and other related traits in wheat, the cumulative contribution from significant *aa* effects (0.3%–16.5%) was small relative to that from *a* effects (3.1%–51.5%) for each trait investigated (Figure 2). Recent studies of yield-associated traits in rice and barley also showed that many epistatic effects were significant, but were all likewise small in magnitude relative to the additive effects [25], [32], [35]. The low percentage of phenotypic variance explained by epistatic effects is apparently due to a large number of QTLs with small effects.

The genotype-by-environment interaction is important in determining the adaptation and fitness of genotypes in the physical environment [32]. Numerous cases of such interactions have been documented [8], [36]. In our study the DH population was evaluated in five locations over one to seven years with two water regimes. A few additive QTLs (for yield per plant (YP), number of spikes per plant (NSP), total number of spikelets per spike (TNSS), proportion of fertile spikelets per spike (PFSS), spike length (SL) and plant height (PH)) and epistatic interactions (for number of grains per spike (NGS), 1000-grain weight (TGW), number of sterile spikelets per spike (NSSS) and PFSS), and more than half the additive QTLs (for NGS, TGW and NSSS) and epistatic interactions (for YP and NSP) were involved in interactions with environments. QTL by year × location (YL) interaction effect was the major source of environmental interaction by further QTL mapping to detect *QE* interaction effects, including QTL by YL interaction under the same water regime, or QTL by water regime interaction under the same YL combination (data not shown). Several desirable QTLs detected under year × location × drought stress (DS) combination, such as *QNGS.cgb-2B*, *QTNSS.cgb-2D.1* and *QPH.cgb-6B.6*, were identified with the consistent effects in the same year × location combinations under DS. But no QTL by water regime interactions were detected under the same YL combination, except that one QTL controlling YP on 2D (near *Xwmc* 453.1) was detected with negative *ae* effect (−0.57^***^) in hd99 (Haidian, Beijing in 1999). This indicated that water treatment contributed little to environmental variation in our study. The actual contributions of *ae* and/or *aae* interactions were small compared to *a* effects (Figure 2). Such an outcome is not only ideal for marker-assisted breeding but should facilitate their cloning in situations such as drought-prone environments [37]. In the present study, twenty six major QTLs tagged by 19 flanking markers were identified with little or no environmental interactions, and contributing large phenotypic variation explained (PVE) (Table 8). For these QTLs, the increase in yield and yield-associated trait values ranged from 1.6% to 10.9% over the population mean. These QTLs could be the targets for marker assisted selection in wheat improvement. In our DH population, phenology effects were not considered owing to only 4 day difference at flowering date, 2–3 day difference at maturing date of 150 lines. The identification of QTLs may not be largely confounded by the low range of ‘pheno-environments’[38].

Consistent with other studies [39], QTLs governing grain yield and yield-associated traits in the present work were distributed on chromosomes in a non-random manner. The most QTLs (22) were identified on 2D, whereas only 2 QTLs were on 4D. Although no QTL was found on 6D in our study, QTLs controlling grain yield and yield components (NSP, TGW, NGS) were identified on that chromosome in other studies [28]–[30], [36], [40]. Failure to detect a QTL in our case might be because we had fewer markers on 6D, there was no segregation for trait differences involving that chromosome or the effect of a QTL was too small in a cross in which many other stronger QTLs were identified.

QTLs for yield-associated traits were likewise highly concentrated in a few chromosomal regions on the same chromosomes (Figure 1). These QTL clusters were generally involved in correlated traits. Similar associations were found in other grain yield component studies [6], [7], [9], [13], [14], [28], [36], [40]. Clustered QTLs for YP, NSP and SL and sometimes NGS and TGW in the same region of chromosome 1B were reported by Börner et al. (2002), Huang et al. (2003), Kumar et al. (2007), Quarrie et al. (2005) and Wang et al. (2009) [7], [11], [14], [28], [36]. All traits except NSSS and PFSS in yield-associated QTL clusters on 2B were observed by Habash et al. (2007), Huang et al. (2006), Kumar et al. (2007), Maccaferri et al. (2008) and McCartney et al. (2005) [13], [21], [29], [30], [36]. The yield related QTL cluster on 4B was near the green revolution gene *Rht-B1* locus and YP, NSP, NGS and TGW QTLs were also detected by others [12]–[14], [29], [30], [36]. The NSSS and PFSS QTLs concerning spikelet fertility in the above three QTL clusters on chromosome 1B, 2B and 4B were not reported previously. As the favorable alleles of the additive QTLs in these three clusters were from the same parents, we speculate that the clusters were preserved during long-term selection for grain yield, and we believed that they are of importance for grain yield determination. Additionally, we detected a yield QTL adjacent to a SL QTL and a QTL cluster involved in NGS, NSSS, PFSS and DSS on 7A. In other studies, not only were QTLs for YP, NGS and fertility-related traits detected, but they were associated with QTLs for NSP, TGW, TNSS and PH in the same bin [2], [13], [14], [29], [36], [41]. This region appears to be important for grain yield because the favorable alleles of the additive QTLs were all from Lumai 14 and the majority of QTLs had strong effects. In addition, the yield-related QTL cluster (YP, SL and TNSS), with two clusters (NGS and PH) and (TGW, TNSS, SL and PH), was located in the centromere region of chromosome 2D YP, SL and TGW QTLs were detected in other studies [2], [13], [36], [41]. The region *Xwmc231-3B* - *Xgwm644.2-3B* having epistatic QTLs for YP, TGW, SL and PH were found to have main-effect QTLs controlling YP, TGW, NGS, NSP in other studies [2], [12], [28], [29], [36]. Based on these QTL clusters, it seems likely that such QTL clusters associated with yield are determinants of biomass [14], and that each yield component is determined at a different phase of plant development [42].

It is well-known that the reduced height gene (*Rht*), vernalization gene (*Vrn*) and photoperiod sensitivity gene (*Ppd*) have been the focus of yield breeding for many years as a means to reduce the height of the crop and to better adapt to their environments by flowering at the appropriate time [14]. Therefore, it is not surprising to observe yield-related QTL clusters on 2B chromosome with *Ppd-B1*, 2D with *Ppd-D1* and *Rht8*, and 4B with the green revolution gene *Rht1* in our study. *Ppd-B1* has been mapped 6.6 cM distal of *Xgwm429* on 2B, and *Ppd-D1* 14.4 cM proximal of *Xgwm261* near *Rht8* on 2D [14]. *Rht1* was mapped near marker *Xgwm165.1* on 4B [14].

Additionally, corresponding with the location of the vernalisation gene *Vrn-A1* on 5A [14], two yield component related QTL clusters (TGW, NGS and PFSS) and (TNSS, NSSS, PFSS and PH) were located; TGW and TNSS were reported by others [25], [36], [41]. On chromosome 3A distributed an earliness *per se* gene *Eps-A1* affecting the plant development [7], two distinct yield component related QTL clusters (TGW, NGS and PH) and (NSP, NGS, NSSS, DSS and PFSS) were anchored in the same bin; TGW and NGS QTLs were also detected by others [4], [28], [29]. The presence of NSSS and PFSS QTLs with large *a* effects in the two regions on 3A and 5A may indicate the genes affecting yield component such as TGW and/or NGS by influencing spikelet sterility. Lastly, it should be mentioned that we identified a QTL cluster (*Xwmc269.3-6B* - *P4232.1-6B*) involving QTLs for NSP, TGW and PH with strong *a* effects, which appears to be new.

Because yield *per se* is a complex trait with low heritability, breeding high yielding wheat cultivars by direct selection for yield has generally been slow [2]. The coincidence of yield QTL with that of at least one yield component with high heritability offers a means for selecting for grain yield by efficient selection for one component [2]. High heritabilities of the yield components were also observed in our study (Table 3). Additionally, due to genetic variation in yield and yield associated traits results from allelic segregations of many QTLs, those QTLs with large additive effects might result in larger difference in the traits of interest, which are valuable for breeders [43]. Further, it indicated an altered frequency of QTL alleles consistent with the agronomic data that the high-yielding progeny lines had an increased frequency of QTL for increased grain yield, grain number, grain weight, total number of spikelets, proportion of fertile spikelets, spike length, plant height and fewer sterile spikelets and spikelets density than the low grain yielding progeny. The present results exhibited that potential improvement in yield and yield components could be made by selecting for some of the identified QTLs. These predictions should be validated by marker assisted selection for the identified component QTL followed by yield assessments.

### Conclusions

QTL analysis showed that though yield-associated traits were subjected to additive main effects, epistatic main effects and their environmental modifications, the additive main-effect QTLs were the major genetic component. All chromosomes except 6D were observed with QTLs controlling yield-associated traits, but among different chromosomes QTLs were non-randomly distributed. A great number of QTLs were located on chromosome 1B, 2B, 2D, 5A and 6B. QTL cluster was another general characteristic of QTL distribution strikingly delegated by three yield-related QTL clusters on 1B, 2B and 4B which accounted for the correlations between YP and other traits well. Twenty six major QTLs with large phenotypic variation explained (PVE) could be the targets for marker assisted selection in wheat improvement. A small group high-yielding progeny was derived by selecting two major yield components TGW and NGS, which was rich in QTLs for higher YP, NGS, TGW, TNSS, PFSS, SL, PH and fewer NSSS than the low yielding progeny, indicating the potential of marker-assisted selection to facilitate wheat production later.

## Materials and Methods

### Ethics Statement

We have obtained the relevant permission for our field studies for growing the DH population and parents in three locations in China over 1 year from the corresponding institutions. They are the Luoyang Academy of Agricultural Sciences in Luoyang (ly) Henan, the Northwest Agriculture & Forest University in Fuping (fp) Shaanxi, and the Institute of Crop Science, Shanxi Academy of Agricultural Sciences in Fenyang (fy) Shanxi.

### Plant materials

A doubled haploid population of 150 lines was developed by microspore culture from the F_1_ hybrid of Hanxuan 10 and Lumai 14, two Chinese common wheat cultivars [44]. Hanxuan 10, a drought-tolerant cultivar from the Shanxi Academy of Agricultural Sciences, released in 1966, was still sporadically grown in arid and barren areas during the past decade. Lumai 14, a high-yielding cultivar adapted to abundant water and fertile conditions, was developed at the Yantai Institute of Agricultural Sciences, Shandong, and was widely grown in northern China during the 1990s. The phenology characteristics of the DH lines and their parents are similar. Flowering date of Hanxuan 10 is one day earlier than that of Lumai 14. The flowering date range of 150 DH lines is 4 day.

### Field trials

The DH population and parents were grown in five locations in China over 1, 2 and 7 years providing data for 12 year-location combinations (Table 11). The five locations include the experimental stations of the Institute of Crop Sciences, Chinese Academy of Agricultural Sciences in Haidian (hd) and in Changping (cp) Beijing, experiment stations of the Luoyang Academy of Agricultural Sciences in Luoyang (ly) Henan, the Northwest Agriculture & Forest University in Fuping (fp) Shaanxi, and the Institute of Crop Science, Shanxi Academy of Agricultural Sciences in Fenyang (fy) Shanxi. They are distributed in the different regions of northern China, including winter zone (Haidian and Changping in Beijing, Fenyang Shanxi) and facultative wheat zone (Luoyang Henan, and Fuping Shaanxi). They are characterized in terms of temperature, radiation, moisture and so on. For the nearest two locations in Beijing, wheat maturity in Haidian Beijing is 3–4 days earlier than in Changping Beijing generally because of the different temperature and rainfall. The 12 year-location trials were unevenly executed. Namely, most of 7 trials were conducted in Haidian Beijing, 2 trials were in Changping Beijing and 1 trial was in other three locations, only one year (1999–2000) with the most of three trials. We have obtained the relevant permission for our field studies for growing the DH population and parents in three locations in China over 1 year from the corresponding institutions. They are the Luoyang Academy of Agricultural Sciences in Luoyang (ly) Henan, the Northwest Agriculture & Forest University in Fuping (fp) Shaanxi, and the Institute of Crop Science, Shanxi Academy of Agricultural Sciences in Fenyang (fy) Shanxi.

**Table 11:** Latitudes and longitudes of five locations and growing season rainfalls in each year × location combination.

Locations^1^	Years							
	(1998–1999)	(1999–2000)	(2000–2001)	(2001–2002)	(2003–2004)	(2004–2005)	(2005–2006)	(2006–2007)
Haidian (hd)	hd98	hd99	hd00		hd03	hd04	hd05	hd06
(116°28′E; 39°48′N)	(162.0 mm)^2^	(143.0 mm)	(202.0 mm)		(146.0 mm)	(141.6 mm)	(100.0 mm)	(124.0 mm)
Changping (cp)							cp05	cp06
(116°13′E; 40°13′N)							(100.1 mm)	(123.1 mm)
Luoyang (ly)		ly99						
(112°14′E; 34°75′N)		(170.0 mm)						
Fuping (fp)		fp99						
(109°17′E; 34°76′N)		(180.0 mm)						
Fenyang (fy)				fy01				
(111°75′E; 37°27′N)				(122.8 mm)				

1 Haidian (hd): Haidian, Beijing; Changping (cp): Changping, Beijing; Luoyang (ly): Luoyang, Henan; Fuping (fp): Fuping, Shaanxi; Fenyang (fy): Fenyang, Shanxi. They are the same meaning hereinafter;

2 The growing season rainfalls from sowing (∼ October 1st) to harvest (∼ next June 20th).

In each year-location combination (YL), two water regimes were applied as drought stressed (DS) and well-watered (WW). DS treatments were represented by rain-fed conditions. The rainfalls are shown in Table 11. WW treatments were irrigated with 750 m^3^/ha four times: at the pre-overwintering, jointing, flowering and grain filling stages, respectively. All the DH lines, along with the parents were planted in Haidian (Beijing) in two-row plots with a length of 2 m and 30 cm spacing, whereas all lines were grown in four-row plots with a length of 4 m and 30 cm spacing in other locations. The field management followed standard agricultural practices. A total of 24 environments of year × location × treatments combination were investigated.

### Trait evaluation

At maturity, five plants in the middle of each plot were randomly sampled for analysis. Eight traits (Table 12), including YP, NSP, NGS, TGW, TNSS, NSSS, SL and PH, were measured. Two derived traits, PFSS and DSS, were calculated as described in Table 12. For some external reasons, these traits were phenotyped in 14 to 23 environments (Table 12).

**Table 12:** Yield-associated traits with their environments evaluated in wheat DH lines.

Traits	Abbreviations	Environments evaluated	Methods of measurement
Yield per plant	YP	hd98^1^, hd99, ly99, fp99, hd00, hd03, hd05	Mass of grain harvested per plant (g)
Number of spikes per plant	NSP	hd98, hd99, ly99, fp99, hd00, hd03, hd04, hd05	Average number of spikes per plant
Number of grains per spike	NGS	hd98, hd99, ly99, fp99, hd00, fy01(none in WW), hd03, hd05	Average number of kernel per spike
1000-grain weight	TGW	hd98, hd99, ly99, fp99, hd00, fy01(none in WW), hd03, hd04, hd05, cp05, hd06, cp06	Mass of a 1000-kernel sample (g)
Total number of spikelets per spike	TNSS	hd98, hd99, ly99, fp99, hd00, fy01, hd03, hd04, hd05	Average number of spikelets per spike
Number of sterile spikelets per spike	NSSS	hd98, hd99, ly99, fp99, hd00, fy01, hd03, hd04, hd05	Average number of sterile spikelets per spike
Proportion of fertile spikelets per spike	PFSS	hd98, hd99, ly99, fp99, hd00, fy01, hd03, hd04, hd05	TNSS subtract NSSS divided by TNSS
Spike length	SL	hd98, hd99, ly99, fp99, hd00, fy01, hd03, hd04, hd05	Average length per spike (cm)
Density of spikelets per spike	DSS	hd98, hd99, ly99, fp99, hd00, fy01, hd03, hd04, hd05	TNSS divided by SL (number of spikelets per cm spike)
Plant height	PH	hd98, hd99, ly99, fp99, hd00, fy01, hd03, hd04, hd05, hd06, cp06	Average plant height measured from the soil surface to tip of spike (cm)

1 hd98 mean Haidian, Beijing (hd) in 1998 under drought stressed (DS) and well-watered (WW) treatment, other similar.

### Statistical analysis

Statistical analysis was implemented using the SAS V8.0 statistics package (SAS Institute Inc., 1999). Pearson's correlation coefficients between pairs of traits were determined for each year × location × water regime environments and mean environment using the ‘proc corr’ procedure. The ANOVA-general linear model (GLM) analysis was performed to determine the significances of differences between DH line genotypes and between year × location × water regime environments. The broad sense heritability of each trait was determined as the ratio of genotypic variance to the sum of the genotypic and environmental variances. To further reveal the contribution of water treatment to phenotypic variation, ANOVA-GLM was carried out to determine the significances of differences between the genotypes, between year-location combinations, between water regimes, and between two-factor combinations (two-factor interactions) for genotypes, year-location combinations and water regimes. Year-location combination was considered as a separate factor because the two factors are not orthogonal (some years have only one location and vice versa).

### QTL analysis

The available genetic linkage map, established from the 150 DH lines using MAPMAKER/Exp version 3.0 software under Kosambis mapping function, consisted of 395 marker loci (132 amplified fragment length polymorphisms (AFLP) and 263 simple sequence repeats (SSR)) covering 3,904 cM with an average distance of 9.9 cM between adjacent markers [45]–[47]. QTL analysis was performed on line values for each trait. QTL detection was undertaken using the mixed linear composite interval mapping model in QTLNetwork 2.0 [23], [48]. In the mixed linear model, the phenotypic value of the *k*-th DH line in environment *h* (*y_hk_*) can be expressed as the following:
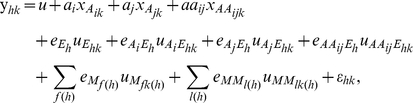

*μ* is the population mean; *a_i_* and *a_j_* are the additive effects (fixed effects) of two putative loci *Q_i_* and *Q_j_*, respectively; *aa_ij_* is the additive × additive epistatic effect (fixed effect) between the two loci; 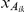, 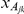 and 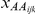 are the coefficients of these genetic main effects; 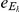 is the random effect of environment *h* with a coefficient 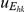; 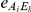 (or 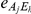) is the random additive × environment interaction effect with a coefficient 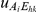 (or 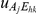) for *Q_i_* (or *Q_j_*); 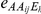 is the random epistasis × environment interaction effect with a coefficient 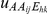; 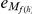 is the random effect of marker *f* nested within the *h*-th environment with a coefficient 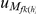, 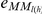 is the random effect of the *l*-th marker × marker interaction nested within the *h*-th environment with a coefficient 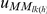; 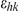 is the random residual effect. The marker factors 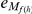 and 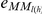 in the model are used to absorb additive and epistatic effects of background QTLs for controlling the noise.

Composite interval analysis was carried out using forward-backward stepwise, multiple linear regression with a probability into and out of the model of 0.05 and a window size set at 10 cM. Significant thresholds for QTL detection were calculated for each dataset using 1,000 permutations and a genome-wide error rate of 0.05. The final genetic model incorporated significant main additive (*a*) and additive × additive epistatic genetic effects (*aa*) and their interactions with environment (*ae* and *aae*). In such a model, all possible pairs of markers were tested.

QTL frequency was estimated for different traits in 41 high-yielding lines (25% selection intensity), including having either high TGW and high NGS (Hgw_Hgn) (four lines), high TGW but low NGS (Hgw_Lgn) (thirteen lines), low TGW but high NGS (Lgw_Hgn) (fifteen lines), or low TGW and low NGS (Lgw_Lgn) (nine lines) as described in Rattey et al. (2009) [49] and McIntyre et al. (2010) [18]. Each putative QTL was represented by its nearest marker, and the frequency of its desirable allele was calculated in the four groups of lines.

## Supporting Information

File S1 **Phenotypic values of yield-associated traits in the wheat parents and DH Lines in different environments.**
(DOC)

File S2 **QTLs affecting yield-associated traits of wheat in different environments.** This is the original QTL mapping results for ten yield-associated traits of wheat in more than 14 year × location × water regime environments.
(DOC)
