# Supplementary material for: Genetic Insight into Yield-Associated Traits of Wheat Grown in Multiple Rain-Fed Environments
Source: PLoS One. 2012 Feb 17;7(2):e31249. doi: 10.1371/journal.pone.0031249 (PMC3281929; doi:10.1371/journal.pone.0031249)
Supplement: File S1 — Phenotypic values of yield-associated traits in the wheat parents and DH Lines in different environments. (DOC) [file pone.0031249.s001.doc]

**File S1** Phenotypic values of yield-associated traits in the wheat parents and DH Lines in different environments

| Trait ^1^ | Item | hd98 ^2^ | | hd99 | | ly99 | | fp99 | | hd00 | | fy01 | | hd03 | | hd04 | | hd05 | | cp05 | | hd06 | | cp06 | |
| --- | --- | --- | --- | --- | --- | --- | --- | --- | --- | --- | --- | --- | --- | --- | --- | --- | --- | --- | --- | --- | --- | --- | --- | --- | --- |
|  |  | DS ^3^ | WW | DS | WW | DS | WW | DS | WW | DS | WW | DS | WW | DS | WW | DS | WW | DS | WW | DS | WW | DS | WW | DS | WW |
| YP | Hanxuan 10 | 14.2 | 11.7 | 14.7 | 17.6 | - ^4^ | - | - | - | 4.5 | 6.4 | - | - | 9.3 | 9.5 | - | - | 8.1 | 11.2 | - | - | - | - | - | - |
|  | Lumai 14 | 12.9 | 14.1 | 11.1 | 12.5 | 15.8 | 18.8 | - | - | 3.1 | 4.8 | - | - | 12.0 | 11.8 | - | - | 6.8 | 9.4 | - | - | - | - | - | - |
|  | Mean | 13.5 | 12.7 | 10.7 | 14.4 | 13.5 | 15.0 | 3.7 | 5.4 | 3.6 | 5.3 | - | - | 9.9 | 8.9 | - | - | 5.8 | 9.0 | - | - | - | - | - | - |
|  | CV ^5^ | 0.2 | 0.2 | 0.2 | 0.2 | 0.2 | 0.2 | 0.2 | 0.2 | 0.2 | 0.2 | - | - | 0.2 | 0.2 | - | - | 0.3 | 0.3 | - | - | - | - | - | - |
|  | Min. | 5.5 | 6.3 | 6.6 | 8.5 | 6.7 | 8.2 | 1.3 | 1.9 | 1.3 | 1.9 | - | - | 4.5 | 4.6 | - | - | 1.2 | 4.0 | - | - | - | - | - | - |
|  | Max. | 21.3 | 22.1 | 15.0 | 24.0 | 21.7 | 22.7 | 6.1 | 8.1 | 5.5 | 8.7 | - | - | 15.6 | 15.5 | - | - | 11.3 | 19.8 | - | - | - | - | - | - |
| NSP | Hanxuan 10 | 10.5 | 11.2 | 9.4 | 11.3 | - | - | - | - | 5.0 | 7.1 | - | - | 7.0 | 8.3 | 8.6 | 8.7 | 8.1 | 8.6 | - | - | - | - | - | - |
|  | Lumai 14 | 6.4 | 7.5 | 5.9 | 7.2 | 8.0 | 8.9 | 4.6 | 6.0 | 2.8 | 4.2 | - | - | 5.5 | 6.5 | 6.7 | 6.7 | 7.3 | 7.6 | - | - | - | - | - | - |
|  | Mean | 10.4 | 10.7 | 7.8 | 10.4 | 9.1 | 9.8 | 5.2 | 7.0 | 4.4 | 6.2 | - | - | 7.3 | 7.8 | 7.7 | 9.2 | 6.9 | 8.7 | - | - | - | - | - | - |
|  | CV | 0.2 | 0.2 | 0.2 | 0.2 | 0.1 | 0.1 | 0.2 | 0.2 | 0.2 | 0.2 | - | - | 0.2 | 0.1 | 0.2 | 0.2 | 0.2 | 0.2 | - | - | - | - | - | - |
|  | Min. | 6.9 | 5.9 | 5.0 | 6.6 | 6.6 | 7.0 | 3.6 | 3.6 | 2.9 | 3.6 | - | - | 4.2 | 5.4 | 3.8 | 6.2 | 2.2 | 5.2 | - | - | - | - | - | - |
|  | Max. | 17.1 | 19.3 | 11.4 | 16.8 | 12.1 | 12.7 | 8.7 | 10.8 | 6.8 | 9.6 | - | - | 10.6 | 9.6 | 12.4 | 14.2 | 11.6 | 15.4 | - | - | - | - | - | - |
| NGS | Hanxuan 10 | 32.9 | 29.0 | 34.3 | 35.4 | - | - | - | - | 25.3 | 24.1 | 18.9 | - | 28.3 | 27.3 | - | - | 23.9 | 38.7 | - | - | - | - | - | - |
|  | Lumai 14 | 43.6 | 42.0 | 40.3 | 43.5 | 43.4 | 49.9 | 31.0 | 33.3 | 33.0 | 31.8 | 17.4 | - | 38.8 | 40.7 | - | - | 30.2 | 37.9 | - | - | - | - | - | - |
|  | Mean | 32.9 | 32.3 | 32.8 | 36.1 | 36.1 | 38.7 | 24.5 | 26.5 | 25.9 | 25.0 | 15.4 | - | 31.2 | 29.7 | - | - | 25.5 | 33.7 | - | - | - | - | - | - |
|  | CV | 0.1 | 0.2 | 0.1 | 0.1 | 0.1 | 0.1 | 0.2 | 0.2 | 0.1 | 0.1 | 0.2 | - | 0.2 | 0.2 | - | - | 0.2 | 0.2 | - | - | - | - | - | - |
|  | Min. | 20.6 | 17.9 | 23.8 | 27.0 | 24.5 | 26.2 | 7.5 | 13.2 | 18.8 | 16.2 | 3.3 | - | 20.9 | 20.5 | - | - | 14.1 | 16.5 | - | - | - | - | - | - |
|  | Max. | 44.9 | 45.1 | 41.2 | 48.6 | 48.4 | 49.4 | 38.6 | 40.1 | 47.1 | 36.4 | 22.6 | - | 49.5 | 42.1 | - | - | 39.5 | 75.1 | - | - | - | - | - | - |
| TGW | Hanxuan 10 | 41.1 | 36.0 | 45.5 | 44.1 | - | - | - | - | 35.2 | 37.4 | - | - | 46.6 | 42.1 | 38.9 | 39.7 | 38.6 | 33.9 | 28.4 | 29.7 | 34.7 | 28.2 | 33.0 | 25.7 |
|  | Lumai 14 | 46.1 | 45.0 | 46.8 | 39.9 | 45.6 | 42.3 | - | - | 33.3 | 35.9 | - | - | 55.0 | 44.4 | 40.6 | 31.2 | 31.8 | 32.7 | 29.7 | 26.7 | 34.6 | 25.1 | 33.2 | 32.3 |
|  | Mean | 39.8 | 37.7 | 42.3 | 39.0 | 41.5 | 39.9 | 29.0 | 29.4 | 32.0 | 34.9 | 34.6 | - | 45.6 | 39.8 | 35.6 | 31.7 | 33.2 | 31.4 | 28.3 | 27.0 | 31.1 | 27.7 | 33.0 | 29.2 |
|  | CV | 0.2 | 0.2 | 0.1 | 0.2 | 0.1 | 0.1 | 0.1 | 0.1 | 0.2 | 0.2 | 0.1 | - | 0.1 | 0.2 | 0.2 | 0.2 | 0.1 | 0.2 | 0.1 | 0.2 | 0.2 | 0.3 | 0.1 | 0.2 |
|  | Min. | 21.9 | 22.0 | 30.7 | 25.4 | 32.3 | 29.8 | 17.8 | 18.8 | 13.4 | 20.1 | 27.3 | - | 30.1 | 23.6 | 21.9 | 20.3 | 24.2 | 18.6 | 19.2 | 17.0 | 17.3 | 12.7 | 22.4 | 15.2 |
|  | Max. | 53.2 | 52.2 | 51.5 | 51.9 | 50.9 | 49.1 | 37.6 | 38.7 | 43.3 | 49.0 | 42.1 | - | 74.1 | 58.6 | 48.0 | 46.0 | 45.9 | 45.2 | 36.7 | 37.8 | 47.8 | 46.7 | 43.0 | 42.4 |
| TNSS | Hanxuan 10 | 16.7 | 17.3 | 17.5 | 17.6 | - | - | - | - | 16.4 | 16.5 | 15.1 | 15.2 | 16.3 | 16.5 | 16.6 | 17.3 | 16.0 | 17.3 | - | - | - | - | - | - |
|  | Lumai 14 | 16.9 | 17.8 | 17.2 | 17.9 | 18.6 | 19.3 | 16.7 | 18.2 | 16.4 | 16.7 | 14.4 | 14.9 | 16.7 | 16.9 | 16.6 | 17.5 | 16.2 | 17.7 | - | - | - | - | - | - |
|  | Mean | 16.3 | 17.2 | 16.8 | 17.1 | 18.0 | 18.4 | 16.2 | 17.5 | 16.2 | 16.4 | 14.1 | 14.5 | 16.3 | 16.3 | 16.2 | 17.0 | 15.3 | 17.1 | - | - | - | - | - | - |
|  | CV | 0.1 | 0.1 | 0.1 | 0.1 | 0.1 | 0.1 | 0.1 | 0.1 | 0.1 | 0.1 | 0.1 | 0.1 | 0.1 | 0.1 | 0.1 | 0.1 | 0.1 | 0.1 | - | - | - | - | - | - |
|  | Min. | 13.5 | 14.4 | 14.7 | 14.8 | 15.3 | 15.7 | 13.7 | 15.1 | 13.9 | 13.9 | 12.0 | 12.4 | 14.0 | 14.0 | 13.4 | 14.6 | 12.2 | 14.7 | - | - | - | - | - | - |
|  | Max. | 19.2 | 19.9 | 19.8 | 19.7 | 20.7 | 21.2 | 18.1 | 20.2 | 18.9 | 19.5 | 16.4 | 16.7 | 19.1 | 19.2 | 18.7 | 19.5 | 17.3 | 20.2 | - | - | - | - | - | - |
| NSSS | Hanxuan 10 | 1.5 | 1.8 | 1.8 | 1.5 | - | - | - | - | 2.5 | 2.5 | 4.6 | 4.0 | 1.8 | 2.0 | 2.7 | 3.3 | 2.1 | 1.4 | - | - | - | - | - | - |
|  | Lumai 14 | 0.9 | 1.8 | 1.4 | 1.4 | 1.4 | 1.2 | 2.1 | 2.5 | 1.7 | 1.8 | 4.0 | 3.2 | 1.3 | 0.9 | 3.2 | 2.9 | 2.0 | 1.4 | - | - | - | - | - | - |
|  | Mean | 1.8 | 2.4 | 2.2 | 1.7 | 2.1 | 2.0 | 3.2 | 4.0 | 2.7 | 2.7 | 4.5 | 3.9 | 2.1 | 2.0 | 3.3 | 3.4 | 2.2 | 2.0 | - | - | - | - | - | - |
|  | CV | 0.4 | 0.4 | 0.3 | 0.4 | 0.3 | 0.3 | 0.3 | 0.3 | 0.3 | 0.4 | 0.2 | 0.2 | 0.4 | 0.4 | 0.3 | 0.4 | 0.4 | 0.5 | - | - | - | - | - | - |
|  | Min. | 0.5 | 0.8 | 0.6 | 0.5 | 0.2 | 0.6 | 1.7 | 1.5 | 0.7 | 1.1 | 2.7 | 1.9 | 0.4 | 0.5 | 1.2 | 0.8 | 0.4 | 0.4 | - | - | - | - | - | - |
|  | Max. | 5.1 | 6.4 | 6.4 | 5.2 | 5.2 | 3.6 | 9.5 | 9.4 | 5.3 | 7.0 | 10.3 | 6.5 | 5.6 | 4.9 | 9.6 | 8.7 | 7.6 | 6.3 | - | - | - | - | - | - |
| PFSS | Hanxuan 10 | 0.9 | 0.9 | 0.9 | 0.9 | - | - | - | - | 0.8 | 0.8 | 0.7 | 0.7 | 0.9 | 0.9 | 0.8 | 0.8 | 0.9 | 0.9 | - | - | - | - | - | - |
|  | Lumai 14 | 0.9 | 0.9 | 0.9 | 0.9 | 0.9 | 0.9 | 0.9 | 0.9 | 0.9 | 0.9 | 0.7 | 0.8 | 0.9 | 0.9 | 0.8 | 0.8 | 0.9 | 0.9 | - | - | - | - | - | - |
|  | Mean | 0.9 | 0.9 | 0.9 | 0.9 | 0.9 | 0.9 | 0.8 | 0.8 | 0.8 | 0.8 | 0.7 | 0.7 | 0.9 | 0.9 | 0.8 | 0.8 | 0.9 | 0.9 | - | - | - | - | - | - |
|  | CV | 0.0 | 0.1 | 0.0 | 0.0 | 0.0 | 0.0 | 0.1 | 0.1 | 0.1 | 0.1 | 0.1 | 0.1 | 0.1 | 0.1 | 0.1 | 0.1 | 0.1 | 0.1 | - | - | - | - | - | - |
|  | Min. | 0.7 | 0.6 | 0.6 | 0.7 | 0.7 | 0.8 | 0.4 | 0.5 | 0.7 | 0.6 | 0.2 | 0.6 | 0.7 | 0.7 | 0.4 | 0.5 | 0.5 | 0.7 | - | - | - | - | - | - |
|  | Max. | 1.0 | 1.0 | 1.0 | 1.0 | 1.0 | 1.0 | 0.9 | 0.9 | 0.9 | 0.9 | 0.8 | 0.9 | 1.0 | 1.0 | 0.9 | 1.0 | 1.0 | 1.0 | - | - | - | - | - | - |
| SL | Hanxuan 10 | 8.1 | 7.9 | 8.3 | 8.4 | - | - | - | - | 7.9 | 7.2 | 5.8 | 6.3 | 8.2 | 7.7 | 7.6 | 7.9 | 7.6 | 9.0 | - | - | - | - | - | - |
|  | Lumai 14 | 8.8 | 8.7 | 8.5 | 9.0 | 8.8 | 9.3 | 7.9 | 8.7 | 8.7 | 8.3 | 6.4 | 7.1 | 8.5 | 8.5 | 8.0 | 8.7 | 8.1 | 9.3 | - | - | - | - | - | - |
|  | Mean | 8.2 | 8.1 | 8.0 | 8.3 | 8.2 | 8.4 | 7.2 | 8.0 | 7.9 | 7.6 | 5.8 | 6.4 | 8.0 | 7.9 | 7.6 | 7.9 | 7.5 | 8.7 | - | - | - | - | - | - |
|  | CV | 0.1 | 0.1 | 0.1 | 0.1 | 0.1 | 0.1 | 0.1 | 0.1 | 0.1 | 0.1 | 0.1 | 0.1 | 0.1 | 0.1 | 0.1 | 0.1 | 0.1 | 0.1 | - | - | - | - | - | - |
|  | Min. | 6.2 | 5.7 | 5.3 | 5.5 | 5.1 | 5.4 | 4.8 | 5.3 | 5.5 | 5.1 | 4.3 | 4.4 | 5.5 | 5.3 | 5.1 | 5.2 | 5.3 | 6.5 | - | - | - | - | - | - |
|  | Max. | 10.5 | 10.6 | 10.9 | 10.9 | 11.5 | 11.6 | 10.1 | 10.6 | 10.5 | 9.9 | 7.7 | 8.6 | 10.5 | 10.6 | 10.2 | 10.8 | 10.0 | 11.5 | - | - | - | - | - | - |
| DSS | Hanxuan 10 | 2.1 | 2.2 | 2.1 | 2.1 | - | - | - | - | 2.1 | 2.3 | 2.6 | 2.4 | 2.0 | 2.1 | 2.2 | 2.2 | 2.1 | 1.9 | - | - | - | - | - | - |
|  | Lumai 14 | 1.9 | 2.1 | 2.0 | 2.0 | 2.1 | 2.1 | 2.1 | 2.1 | 1.9 | 2.0 | 2.3 | 2.1 | 2.0 | 2.0 | 2.1 | 2.0 | 2.0 | 1.9 | - | - | - | - | - | - |
|  | Mean | 2.0 | 2.1 | 2.1 | 2.1 | 2.2 | 2.2 | 2.3 | 2.2 | 2.1 | 2.2 | 2.4 | 2.3 | 2.1 | 2.1 | 2.1 | 2.2 | 2.1 | 2.0 | - | - | - | - | - | - |
|  | CV | 0.1 | 0.1 | 0.1 | 0.1 | 0.1 | 0.1 | 0.1 | 0.1 | 0.1 | 0.1 | 0.1 | 0.1 | 0.1 | 0.1 | 0.1 | 0.1 | 0.1 | 0.1 | - | - | - | - | - | - |
|  | Min. | 1.5 | 1.7 | 1.6 | 1.6 | 1.6 | 1.7 | 1.8 | 1.8 | 1.6 | 1.6 | 1.9 | 1.9 | 1.6 | 1.6 | 1.6 | 1.5 | 1.6 | 1.5 | - | - | - | - | - | - |
|  | Max. | 2.8 | 3.1 | 3.1 | 3.2 | 3.5 | 3.4 | 3.5 | 3.4 | 3.0 | 3.3 | 3.1 | 3.3 | 3.0 | 3.0 | 3.2 | 3.3 | 2.9 | 2.7 | - | - | - | - | - | - |
| PH | Hanxuan 10 | 117.8 | 121.9 | - | - | - | - | - | - | 101.4 | 112.1 | 77.0 | 106.5 | 110.4 | 116.3 | 111.0 | 126.4 | 104.8 | 120.5 | - | - | 125.0 | 132.8 | 111.3 | 123.1 |
|  | Lumai 14 | 71.8 | 72.8 | - | - | 66.7 | 73.2 | 54.8 | 58.1 | 63.0 | 69.4 | 56.3 | 81.5 | 66.5 | 71.5 | 68.8 | 79.6 | 68.8 | 78.4 | - | - | 70.9 | 84.5 | 70.0 | 78.1 |
|  | Mean | 91.2 | 95.4 | 79.3 | 89.3 | 86.7 | 96.0 | 60.1 | 69.6 | 82.0 | 87.5 | 61.0 | 87.0 | 86.3 | 92.7 | 86.5 | 99.3 | 79.7 | 96.6 | - | - | 94.7 | 106.4 | 87.7 | 99.1 |
|  | CV | 0.2 | 0.2 | 0.2 | 0.2 | 0.2 | 0.2 | 0.1 | 0.2 | 0.2 | 0.2 | 0.1 | 0.2 | 0.2 | 0.2 | 0.2 | 0.2 | 0.2 | 0.2 | - | - | 0.2 | 0.2 | 0.2 | 0.2 |
|  | Min. | 56.7 | 59.8 | 52.0 | 58.5 | 60.5 | 65.0 | 39.0 | 40.0 | 50.5 | 52.5 | 40.0 | 60.0 | 60.0 | 60.2 | 53.4 | 65.4 | 45.6 | 66.2 | - | - | 53.0 | 60.0 | 54.0 | 62.0 |
|  | Max. | 116.8 | 132.3 | 104.5 | 121.0 | 112.5 | 127.5 | 80.0 | 93.5 | 109.5 | 119.5 | 81.0 | 116.0 | 113.6 | 127.6 | 115.8 | 128.4 | 107.0 | 132.4 | - | - | 125.0 | 141.0 | 117.0 | 133.0 |

^1^ YP (g): Yield per plant; NSP: Number of spikes per plant; NGS: Number of grains per spike; TGW (g): 1000-grain weight; TNSS: Total number of spikelets per spike; NSSS: Number of sterile spikelets per spike; PFSS: Proportion of fertile spikelets per spike; SL (cm): Spike length; DSS: Density of spikelets per spike; PH (cm): Plant height.

^2^ hd98 means Haidian, Beijing in 1998, other similar. ^3^ DS: drought stressed; WW: well-watered. ^4^ “-” indicates missing data. ^5^ CV: coefficient of variation.
