# Supplementary material for: Genetic Insight into Yield-Associated Traits of Wheat Grown in Multiple Rain-Fed Environments
Source: PLoS One. 2012 Feb 17;7(2):e31249. doi: 10.1371/journal.pone.0031249 (PMC3281929; doi:10.1371/journal.pone.0031249)
Supplement: File S2 — QTLs affecting yield-associated traits of wheat in different environments. This is the original QTL mapping results for ten yield-associated traits of wheat in more than 14 year × location × water regime environments. (DOC) [file pone.0031249.s002.doc]

**File S2** **QTLs affecting yield-associated traits of wheat in different environments**

| QTL/QTL_i_ | Near mar,/ Near mar,_i_ | Dis.. ^4^ | QTL_j_ | Near mar._j_ | Dis.. | *a*/*aa* ^5^ | *ae*/*aae* | h^2(*a*)/ h^2(*aa*) (%) | h^2(*ae*)/ h^2(*aae*) (%) |
| --- | --- | --- | --- | --- | --- | --- | --- | --- | --- |
| *QYP.cgb-1B.1* ^1^ | *Xcwm65*  ^3^ | 0.0 |  |  |  | 0.194^**** 6^ | -0.306^**^(hd04T), -0.240^*^(hd98C), 0.255^*^(hd06C) ^7^ | 0.24 | 0.28 |
| *QYP.cgb-2B* | *P5322* | 0.0 |  |  |  | -0.254^****^ |  | 0.25 |  |
| *QYP.cgb-2D* | *Xgwm157* | 4.0 |  |  |  | 0.423^****^ |  | 1.07 |  |
| ***QYP.cgb-3D*** ^2^ | *Xgdm8* | 10.0 |  |  |  | -0.248^****^ | -0.574^****^(hd98T), 0.390^**^(hd06T) | 0.17 | 0.35 |
| ***QYP.cgb-4B.1*** | *-Xgwm513* | 2.5 |  |  |  | -0.237^****^ |  | 0.34 |  |
| *QYP.cgb-5B* | *Xwmc363* | 0.0 |  |  |  | -0.286^****^ | -0.300^**^(ly99T), 0.226^*^(hd06C) | 0.33 | 0.21 |
| *QYP.cgb-7A* | *-P1111* | 1.0 |  |  |  | -0.188^****^ |  | 0.24 |  |
| *QYP.cgb-7D* | *Xgdm86* | 2.0 |  |  |  | -0.275^****^ |  | 0.44 |  |
| *QYP.cgb-1B.2* | *P3477.1* | 4.0 | *QYP.cgb-4B.2* | *Xgwm149* | 2.0 | -0.198^****^ | -0.267^*^(hd98T), -0.341^*^(ly99T), 0.346^*^(hd06C) | 0.10 | 0.23 |
| *QYP.cgb-2A* | *-P3613.1* | 5.9 | *QYP.cgb-3B* | *Xgwm644.2* | 0.0 | 0.199^****^ | 0.341^**^(hd98C) | 0.15 | 0.23 |
| ***QYP.cgb-3D*** | *Xgdm8* | 10.0 | ***QYP.cgb-4B.1*** | *-Xgwm513* | 2.5 | -0.152^***^ |  | 0.06 |  |
| *QNSP.cgb-1B.2* | *Xcwm70* | 0.0 |  |  |  | 0.063^*^ |  | 0.42 |  |
| *QNSP.cgb-2B* | *Xgwm319* | 6.0 |  |  |  | 0.317^****^ | -0.179^*^(hd05C) | 0.92 | 0.42 |
| *QNSP.cgb-4A* | *P3446.2* | 6.0 |  |  |  | -0.377^****^ |  | 0.94 |  |
| *QNSP.cgb-5A* | *-Xgwm154* | 1.4 |  |  |  | 0.188^****^ |  | 0.86 |  |
| *QNSP.cgb-6B* | *Xwmc269.3* | 0.0 |  |  |  | 0.367^****^ | 0.255^***^(hd98T), -0.234^*^(hd06T), 0.177^*^(hd98C), 0.299^***^(hd99C) | 1.40 | 0.51 |
| *QNSP.cgb-7A.1* | *P3454.5* | 0.0 |  |  |  | -0.078^***^ |  | 0.43 |  |
| *QNSP.cgb-1A* | *Xgwm164* | 0.0 | *QNSP.cgb-6A.1* | *-P4232.4* | 1.4 | -0.147^****^ | -0.275^***^(hd98C), -0.165^*^(hd99C) | 0.29 | 0.45 |
| *QNSP.cgb-1B.1* | *Xwmc156* | 0.0 | *QNSP.cgb-7B* | *-P3446.7* | 1.4 | 0.146^****^ | -0.177^*^(hd98T), 0.205^**^(hd06C) | 0.25 | 0.34 |
| *QNSP.cgb-2D* | *Xwmc41* | 0.0 | *QNSP.cgb-3A* | *-Xwmc21* | 1.5 | 0.186^****^ | 0.406^****^(hd98C), -0.165^*^(hd06C) | 0.47 | 0.44 |
| *QNSP.cgb-4B* | *Xgwm513* | 0.0 | *QNSP.cgb-5D.1* | *-Xgdm68* | 1.5 | -0.227^****^ | -0.331^****^(hd98C), -0.260^***^(hd99C), 0.189^*^(hd05C) | 0.54 | 0.59 |
| *QNSP.cgb-4B* | *Xgwm513* | 0.0 | *QNSP.cgb-5D.2* | *Xgdm3* | 20.0 | 0.088^**^ |  | 0.07 |  |
| *QNSP.cgb-6A.2* | *Xwmc179.3* | 0.0 | *QNSP.cgb-7A.2* | *Xgwm282* | 2.0 | 0.248^****^ | 0.151^*^(hd99C) | 0.68 | 0.28 |
| *QNGS.cgb-2B* | *P4233.1* | 0.0 |  |  |  | -0.935^****^ | 0.458^*^(fy01T), 0.727^***^(hd06T) | 2.82 | 0.36 |
| ***QNGS.cgb-2D.1*** | *Xwmc453.1* | 12.0 |  |  |  | 0.850^****^ |  | 2.06 |  |
| ***QNGS.cgb-3A.2*** | *-Xwmc21* | 1.5 |  |  |  | -0.810^****^ | -0.496^**^(hd06C) | 0.43 | 0.25 |
| ***QNGS.cgb-4A*** | *Xcwm145* | 8.0 |  |  |  | 0.786^****^ |  | 1.55 |  |
| *QNGS.cgb-6A* | *Xwmc179.1* | 4.0 |  |  |  | -1.444^****^ |  | 1.73 |  |
| ***QNGS.cgb-6B.4*** | *Xgwm219* | 0.0 |  |  |  | 0.951^****^ |  | 0.38 |  |
| *QNGS.cgb-7A* | *Xwmc488* | 2.0 |  |  |  | -1.682^****^ | -0.596^*^(hd06C) | 2.56 | 0.29 |
| ***QNGS.cgb-7D.1*** | *-Xgdm88* | 9.4 |  |  |  | -0.417^****^ | 1.975^****^(hd06C) | 0.18 | 0.61 |
| ***QNGS.cgb-3A.2*** | *-Xwmc21* | 1.5 | ***QNGS.cgb-4A*** | *Xcwm145* | 8.0 | -0.544^****^ |  | 0.34 |  |
| ***QNGS.cgb-6B.4*** | *Xgwm219* | 0.0 | ***QNGS.cgb-7D.1*** | *-Xgdm88* | 9.4 | 0.662^****^ |  | 0.72 |  |
| *QNGS.cgb-1A.1* | *Xwmc120* | 0.0 | *QNGS.cgb-7B.1* | *Xwmc311* | 0.0 | 0.308^****^ |  | 0.33 |  |
| *QNGS.cgb-1A.2* | *Xcwm517* | 4.0 | *QNGS.cgb-7B.1* | *Xwmc311* | 0.0 | -0.894^****^ |  | 0.55 |  |
| *QNGS.cgb-1A.2* | *Xcwm517* | 4.0 | *QNGS.cgb-7B.2* | *-Xgwm611* | 6.7 | -0.333^***^ |  | 0.01 |  |
| ***QNGS.cgb-2D.1*** | *Xwmc453.1* | 12.0 | *QNGS.cgb-2D.2* | *-P4233.2* | 7.2 | 0.729^****^ | 1.176^***^(fp99C), -1.191^***^(hd06C) | 0.29 | 0.32 |
| *QNGS.cgb-3A.1* | *-Xwmc532* | 9.3 | *QNGS.cgb-7D.2* | *-Xgwm44* | 5.9 | 0.377^****^ |  | 0.38 |  |
| *QNGS.cgb-5A.1* | *-Xgwm595* | 5.0 | *QNGS.cgb-7D.2* | *-Xgwm44* | 5.9 | -0.770^****^ |  | 1.15 |  |
| *QNGS.cgb-5A.2* | *Xwmc410* | 12.0 | *QNGS.cgb-7D.2* | *-Xgwm44* | 7.9 | 0.332^***^ |  | 0.01 |  |
| *QNGS.cgb-5A.2* | *Xwmc410* | 14.0 | *QNGS.cgb-7D.3* | *Xgwm44* | 12.0 | -0.871^****^ | -0.896^*^(hd98C) | 0.05 | 0.16 |
| *QNGS.cgb-6B.1* | *P8444.1* | 0.0 | *QNGS.cgb-6B.2* | *Xgwm132* | 0.0 | 1.085^****^ |  | 1.06 |  |
| *QNGS.cgb-6B.1* | *P8444.1* | 0.0 | *QNGS.cgb-6B.3* | *Xcwm29* | 2.0 | -0.276^***^ |  | 0.02 |  |
| *QTGW.cgb-1B* | *-Xgwm259* | 4.6 |  |  |  | -1.205^****^ | -0.711^***^(hd98T), 0.577^*^(hd00T), 0.581^*^(fy01T), -0.590^*^(hd98C) | 1.80 | 0.36 |
| ***QTGW.cgb-2B.1*** | *P5322* | 0.0 |  |  |  | -1.027^****^ | 0.514^*^(fy01T), 0.430^*^(fp99C), -0.433^*^(hd07C) | 1.61 | 0.51 |
| ***QTGW.cgb-2D.1*** | *Xwmc144* | 0.0 |  |  |  | -0.466^****^ | -0.447^*^(hd98C), | 0.33 | 0.26 |
| ***QTGW.cgb-2D.2*** | *P3470.3* | 4.0 |  |  |  | 1.938^****^ | -0.557^*^(fy01T), 0.791^***^(hd03C), -0.631^*^(cp05C) | 4.90 | 0.37 |
| ***QTGW.cgb-3A.3*** | *-Xwmc532* | 9.3 |  |  |  | 1.185^****^ | -0.806^***^(fy01T), 0.682^*^(hd05C) | 1.65 | 0.35 |
| ***QTGW.cgb-3A.4*** | *-P8422* | 1.8 |  |  |  | -1.376^****^ | 0.462^*^(fp99T), 0.455^*^(fy01T), -0.684^***^(hd03C) | 2.87 | 0.37 |
| ***QTGW.cgb-3B.3*** | *-P2076* | 6.4 |  |  |  | -1.082^****^ | 0.461^*^(fp99T), 0.809^***^(fy01T), -0.536^*^(hd07C) | 1.22 | 0.63 |
| *QTGW.cgb-3D* | *Xgwm341* | 2.0 |  |  |  | -0.399^****^ |  | 1.23 |  |
| ***QTGW.cgb-4B.1*** | *-Xgwm368* | 8.8 |  |  |  | -0.514^****^ |  | 0.21 |  |
| ***QTGW.cgb-5A.4*** | *Xgwm595* | 0.0 |  |  |  | -0.404^****^ | 0.517^*^(hd98T), -0.506^*^(hd06T), 0.742^***^(hd98C), 0.589^*^(cp05C), 0.550^*^(cp06T) | 0.46 | 0.39 |
| *QTGW.cgb-6A* | *Xwmc179.1* | 10.0 |  |  |  | 0.971^****^ |  | 1.30 |  |
| *QTGW.cgb-6B* | *Xwmc269.3* | 0.0 |  |  |  | -1.122^****^ | 0.471^*^(ly99T), 0.498^*^(fp99T), 0.839^***^(fy01T), -0.469^*^(hd99C), 0.711^***^(fp99C), -0.846^***^(hd03C) | 1.87 | 0.48 |
| *QTGW.cgb-7A* | *P3454.5* | 0.0 |  |  |  | 0.563^****^ | -0.386^*^(fy01T) | 0.62 | 0.36 |
| ***QTGW.cgb-2B.1*** | *P5322* | 0.0 | ***QTGW.cgb-3A.4*** | *-P8422* | 1.8 | 0.748^****^ |  | 0.87 |  |
| ***QTGW.cgb-2D.1*** | *Xwmc144* | 0.0 | ***QTGW.cgb-3A.4*** | *-P8422* | 1.8 | 0.286^****^ |  | 0.17 |  |
| ***QTGW.cgb-2D.2*** | *P3470.3* | 4.0 | ***QTGW.cgb-5A.4*** | *Xgwm595* | 0.0 | -0.251^****^ |  | 0.21 |  |
| ***QTGW.cgb-3A.3*** | *-Xwmc532* | 9.3 | ***QTGW.cgb-5A.4*** | *Xgwm595* | 0.0 | -0.917^****^ |  | 1.44 |  |
| ***QTGW.cgb-4B.1*** | *-Xgwm368* | 8.8 | ***QTGW.cgb-5A.4*** | *Xgwm595* | 0.0 | 0.373^****^ |  | 0.83 |  |
| *QTGW.cgb-2B.2* | *Xwmc477* | 0.0 | *QTGW.cgb-3B.5* | *Xgwm340* | 0.0 | -0.951^****^ |  | 0.83 |  |
| *QTGW.cgb-3A.1* | *P3614* | 0.0 | *QTGW.cgb-5A.2* | *-P3616.6* | 2.5 | -0.636^****^ |  | 0.18 |  |
| *QTGW.cgb-3A.2* | *-P3716.2* | 1.9 | *QTGW.cgb-5D* | *-Xgdm68* | 5.5 | -0.516^****^ |  | 0.07 |  |
| *QTGW.cgb-3B.1* | *Xwmc231* | 4.0 | *QTGW.cgb-7B.3* | *-Xgwm68.1* | 10.9 | -0.262^***^ |  | 0.59 |  |
| *QTGW.cgb-3B.2* | *Xpsp3030* | 0.0 | *QTGW.cgb-7B.2* | *-Xgwm297* | 16.8 | 0.366^****^ |  | 0.05 |  |
| *QTGW.cgb-3B.2* | *Xpsp3030* | 0.0 | *QTGW.cgb-7B.3* | *-Xgwm68.1* | 10.9 | -0.936^****^ |  | 0.13 |  |
| ***QTGW.cgb-3B.3*** | *-P2076* | 6.4 | *QTGW.cgb-4B.2* | *Xwmc47* | 0.0 | 0.418^****^ |  | 0.15 |  |
| *QTGW.cgb-3B.4* | *Xwmc326* | 18.0 | *QTGW.cgb-7B.1* | *-P1123.2* | 1.7 | 1.060^****^ |  | 0.62 |  |
| *QTGW.cgb-4A.1* | *Xwmc468* | 0.0 | *QTGW.cgb-4A.2* | *-P3613.2* | 4.6 | 0.358^****^ | 0.589^**^(hd06C) | 0.14 | 0.25 |
| *QTGW.cgb-5A.1* | *P2470* | 0.0 | *QTGW.cgb-5D* | *Xgwm205.2* | 0.0 | 0.415^****^ |  | 0.17 |  |
| *QTGW.cgb-5A.3* | *Xwmc524* | 10.0 | *QTGW.cgb-5B.1* | *Xgwm67* | 0.0 | -0.792^****^ |  | 1.28 |  |
| ***QTGW.cgb-5A.4*** | *Xgwm595* | 0.0 | *QTGW.cgb-5B.2* | *Xgwm335* | 0.0 | -0.404^****^ | 0.546^**^(fy01T) | 0.08 | 0.17 |
| ***QTNSS.cgb-1B.1*** | *P6934.3* | 0.0 |  |  |  | -0.263^****^ | 0.074^*^(fp99T), | 1.69 | 0.18 |
| ***QTNSS.cgb-2A*** | *Xgwm448* | 0.0 |  |  |  | 0.118^****^ | 0.093^*^(fp99T), -0.087^*^(hd03T), 0.091^*^(ly99C), 0.094^*^(fp99C) | 0.09 | 0.30 |
| ***QTNSS.cgb-2D.1*** | *-Xwmc144* | 1.1 |  |  |  | 0.158^****^ | 0.095^*^(ly99T), 0.203^****^(fp99T), 0.156^***^(fy01T), -0.092^*^(hd98C), -0.100^*^(hd00C), -0.099^*^(hd05C), -0.097^*^(hd06C) | 0.85 | 0.46 |
| ***QTNSS.cgb-3B*** | *Xcwm539.1* | 0.0 |  |  |  | 0.217^****^ |  | 1.56 |  |
| ***QTNSS.cgb-4A.1*** | *Xwmc89* | 2.0 |  |  |  | -0.097^****^ |  | 1.04 |  |
| ***QTNSS.cgb-4A.2*** | *-P2078* | 11.1 |  |  |  | 0.147^****^ |  | 2.75 |  |
| ***QTNSS.cgb-4A.3*** | *P3613.2* | 0.0 |  |  |  | 0.301^****^ |  | 1.92 |  |
| ***QTNSS.cgb-4B.1*** | *Xgwm513* | 0.0 |  |  |  | -0.292^****^ | 0.068^*^(fy01T), 0.095^***^(fy01C) | 4.77 | 0.22 |
| *QTNSS.cgb-4B.2* | *-P3459.1* | 12.6 |  |  |  | 0.075^****^ |  | 0.16 |  |
| *QTNSS.cgb-5A* | *Xgwm291* | 4.0 |  |  |  | -0.541^****^ |  | 7.43 |  |
| ***QTNSS.cgb-5B.1*** | *Xwmc380* | 0.0 |  |  |  | 0.153^****^ |  | 0.69 |  |
| ***QTNSS.cgb-5B.2*** | *-P5166.3* | 6.4 |  |  |  | -0.186^****^ |  | 1.00 |  |
| ***QTNSS.cgb-6A.1*** | *Xwmc179.1* | 2.0 |  |  |  | -0.337^****^ |  | 2.59 |  |
| ***QTNSS.cgb-6A.2*** | *-P1832* | 5.6 |  |  |  | 0.193^****^ |  | 0.63 |  |
| *QTNSS.cgb-7B* | *Xgwm297* | 8.0 |  |  |  | -0.184^****^ |  | 0.40 |  |
| ***QTNSS.cgb-7D*** | *Xgdm88* | 0.0 |  |  |  | -0.034^***^ |  | 0.64 |  |
| ***QTNSS.cgb-1B.1*** | *P6934.3* | 0.0 | ***QTNSS.cgb-3B*** | *Xcwm539.1* | 0.0 | -0.121^****^ |  | 0.33 |  |
| ***QTNSS.cgb-1B.1*** | *P6934.3* | 0.0 | ***QTNSS.cgb-6A.1*** | *Xwmc179.1* | 2.0 | 0.075^****^ |  | 0.13 |  |
| ***QTNSS.cgb-2A*** | *Xgwm448* | 0.0 | ***QTNSS.cgb-5B.1*** | *Xwmc380* | 0.0 | 0.062^****^ |  | 0.30 |  |
| ***QTNSS.cgb-2D.1*** | *-Xwmc144* | 1.1 | ***QTNSS.cgb-4B.1*** | *Xgwm513* | 0.0 | -0.112^****^ |  | 0.54 |  |
| ***QTNSS.cgb-2D.1*** | *-Xwmc144* | 1.1 | *QTNSS.cgb-7D* | *Xgdm88* | 0.0 | -0.071^****^ |  | 0.13 |  |
| ***QTNSS.cgb-4A.1*** | *Xwmc89* | 2.0 | ***QTNSS.cgb-6A.2*** | *-P1832* | 5.6 | 0.114^****^ |  | 0.40 |  |
| ***QTNSS.cgb-4A.2*** | *-P2078* | 11.1 | ***QTNSS.cgb-5B.2*** | *-P5166.3* | 6.4 | 0.194^****^ |  | 0.64 |  |
| ***QTNSS.cgb-4A.3*** | *P3613.2* | 0.0 | ***QTNSS.cgb-7D*** | *Xgdm88* | 0.0 | 0.069^****^ |  | 0.52 |  |
| *QTNSS.cgb-1A.1* | *Xwmc20* | 0.0 | *QTNSS.cgb-1B.5* | *-Xgwm259* | 0.6 | 0.076^****^ |  | 0.14 |  |
| *QTNSS.cgb-1A.2* | *Xwmc304* | 0.0 | *QTNSS.cgb-2D.2* | *Xgwm157* | 0.0 | -0.047^****^ |  | 0.11 |  |
| *QTNSS.cgb-1B.2* | *P2043.3* | 0.0 | *QTNSS.cgb-7A* | *-Xgwm635.2* | 3.6 | 0.089^****^ |  | 0.23 |  |
| *QTNSS.cgb-1B.3* | *P1133* | 0.0 | *QTNSS.cgb-2B* | *Xgwm374* | 0.0 | 0.062^****^ |  | 0.09 |  |
| *QTNSS.cgb-1B.4* | *P3616.3* | 4.0 | *QTNSS.cgb-2B* | *Xgwm374* | 0.0 | 0.103^****^ |  | 0.15 |  |
| *QTNSS.cgb-2D.3* | *Xwmc181* | 0.0 | ***QTNSS.cgb-4A.2*** | *Xwmc468* | 0.0 | -0.100^****^ |  | 0.33 |  |
| *QTNSS.cgb-5B.3* | *P2454.2* | 0.0 | *QTNSS.cgb-6B.2* | *-P8966.1* | 1.9 | 0.038^***^ |  | 0.11 |  |
| *QTNSS.cgb-5B.3* | *P2454.2* | 0.0 | *QTNSS.cgb-6B.1* | *Xcwm449* | 2.0 | 0.046^***^ |  | 0.01 |  |
| *QTNSS.cgb-5D* | *Xgdm3* | 0.0 | ***QTNSS.cgb-7D*** | *Xgdm88* | 0.0 | -0.083^****^ |  | 0.21 |  |
| *QNSSS.cgb-1B.2* | *P8222.3* | 0.0 |  |  |  | -0.197^****^ | 0.114^*^(hd03T), 0.116^*^(ly99C), -0.204^****^(fp99C), 0.159^***^(fy01C), -0.198^****^(hd05C) | 0.38 | 0.81 |
| *QNSSS.cgb-2A.2* | *Xcwm276* | 0.0 |  |  |  | 0.048^****^ | -0.079^*^(fp99C), 0.086^*^(hd05C) | 0.16 | 0.36 |
| ***QNSSS.cgb-2B*** | *P4233.1* | 0.0 |  |  |  | 0.132^****^ |  | 1.36 |  |
| *QNSSS.cgb-2D.1* | *-Xwmc41* | 8.8 |  |  |  | 0.097^****^ | 0.105^*^(fy01T), 0.096^*^(hd05C) | 0.33 | 0.26 |
| ***QNSSS.cgb-3A*** | *Xwmc21* | 0.0 |  |  |  | 0.134^****^ | 0.094^*^(fp99C), 0.140^***^(hd00C) | 0.36 | 0.45 |
| ***QNSSS.cgb-4B*** | *Xgwm495* | 0.0 |  |  |  |  | 0.090^*^(fy01T), 0.111^**^(fp99C) |  | 0.36 |
| ***QNSSS.cgb-5A*** | *-Xgwm291* | 6.1 |  |  |  | -0.361^****^ |  | 7.18 |  |
| ***QNSSS.cgb-5B.2*** | *P4138* | 4.0 |  |  |  | -0.174^****^ |  | 0.69 |  |
| *QNSSS.cgb-5D* | *-Xgdm43* | 14.4 |  |  |  | 0.095^****^ |  | 0.87 |  |
| ***QNSSS.cgb-6A.1*** | *Xcwm306* | 8.0 |  |  |  | -0.056^***^ | -0.122^*^(hd05T), 0.104^*^(hd03C) | 0.63 | 0.51 |
| ***QNSSS.cgb-7A.1*** | *P2454.4* | 0.0 |  |  |  | -0.034^**^ | -0.107^*^(fy01T), 0.160^***^(fp99C) | 0.12 | 0.47 |
| ***QNSSS.cgb-7A.2*** | *-Xwmc488* | 1.6 |  |  |  | 0.250^****^ |  | 3.77 |  |
| ***QNSSS.cgb-7D.2*** | *Xgwm44* | 12.0 |  |  |  | -0.052^***^ |  | 0.51 |  |
| ***QNSSS.cgb-2B*** | *P4233.1* | 0.0 | *QNSSS.cgb-6A.1* | *Xcwm306* | 8.0 | -0.111^****^ |  | 0.40 |  |
| ***QNSSS.cgb-3A*** | *Xwmc21* | 0.0 | ***QNSSS.cgb-5B.2*** | *P4138* | 4.0 | -0.146^****^ |  | 1.30 |  |
| ***QNSSS.cgb-4B*** | *Xgwm495* | 0.0 | ***QNSSS.cgb-5B.2*** | *P4138* | 4.0 | -0.114^****^ | -0.116^***^(hd05C) | 0.39 | 0.32 |
| ***QNSSS.cgb-4B*** | *Xgwm495* | 0.0 | ***QNSSS.cgb-6A.1*** | *Xcwm306* | 8.0 | -0.077^****^ | -0.140^***^(fy01T), 0.104^*^(hd03C), 0.106^*^(hd04C) | 0.26 | 0.44 |
| ***QNSSS.cgb-5A*** | *-Xgwm291* | 6.1 | ***QNSSS.cgb-6A.1*** | *Xcwm306* | 8.0 | 0.100^****^ |  | 0.25 |  |
| ***QNSSS.cgb-5B.2*** | *P4138* | 4.0 | ***QNSSS.cgb-7A.1*** | *P2454.4* | 0.0 | -0.112^****^ |  | 0.28 |  |
| ***QNSSS.cgb-5B.2*** | *P4138* | 4.0 | ***QNSSS.cgb-7A.2*** | *-Xwmc488* | 1.6 | 0.073^****^ |  | 0.26 |  |
| ***QNSSS.cgb-6A.1*** | *Xcwm306* | 8.0 | ***QNSSS.cgb-7A.1*** | *P2454.4* | 0.0 | 0.139^****^ |  | 0.44 |  |
| ***QNSSS.cgb-7A.1*** | *P2454.4* | 0.0 | ***QNSSS.cgb-7A.2*** | *-Xwmc488* | 1.6 | -0.131^****^ |  | 0.76 |  |
| ***QNSSS.cgb-7A.1*** | *P2454.4* | 0.0 | ***QNSSS.cgb-7D.2*** | *Xgwm44* | 12.0 | 0.164^****^ |  | 0.44 |  |
| ***QNSSS.cgb-7A.2*** | *-Xwmc488* | 1.6 | ***QNSSS.cgb-7D.2*** | *Xgwm44* | 12.0 | -0.187^****^ |  | 0.97 |  |
| *QNSSS.cgb-1B.1* | *Xgwm131* | 0.0 | *QNSSS.cgb-6A.2* | *-Xwmc179.3* | 1.2 | 0.075^****^ |  | 0.04 |  |
| *QNSSS.cgb-1B.1* | *Xgwm131* | 0.0 | *QNSSS.cgb-5B.1* | *Xgwm234* | 6.0 | -0.054^****^ |  | 0.16 |  |
| *QNSSS.cgb-1B.3* | *-Xcwm548* | 0.9 | *QNSSS.cgb-6A.3* | *Xwmc179.3* | 0.0 | 0.079^****^ |  | 0.16 |  |
| *QNSSS.cgb-2A.1* | *P3715.2* | 0.0 | *QNSSS.cgb-7D.1* | *-Xgwm295* | 2.4 | -0.123^****^ | 0.098^*^(fy01C) | 0.64 | 0.26 |
| *QNSSS.cgb-2D.2* | *Xcwm96.2* | 0.0 | *QNSSS.cgb-7B* | *Xgwm611* | 12.0 | -0.074^****^ |  | 0.14 |  |
| *QNSSS.cgb-6A.4* | *Xgwm169* | 0.0 | *QNSSS.cgb-6B* | *Xcwm449* | 0.0 | -0.159^****^ |  | 0.96 |  |
| *QPFSS.cgb-1B* | *P8222.3* | 0.0 |  |  |  | 0.010^****^ | -0.006^*^(ly99C)，0.016^****^(fp99C)，-0.012^***^(fy01C)，0.013^****^(hd04C) | 0.10 | 0.79 |
| *QPFSS.cgb-2B* | *P4233.1* | 0.0 |  |  |  | -0.011^****^ |  | 1.33 |  |
| ***QPFSS.cgb-3A*** | *Xwmc21* | 0.0 |  |  |  | -0.008^****^ |  | 0.25 |  |
| *QPFSS.cgb-5A.1* | *Xwmc524* | 4.0 |  |  |  | 0.007^****^ |  | 1.86 |  |
| *QPFSS.cgb-5A.2* | *-Xgwm291* | 6.1 |  |  |  | 0.014^****^ |  | 2.59 |  |
| ***QPFSS.cgb-5B*** | *P4138* | 0.0 |  |  |  | 0.005^****^ | 0.007^*^(fy01C)，-0.006^*^(hd03C), 0.006^*^(hd04C) | 0.81 | 0.56 |
| *QPFSS.cgb-6A* | *P3474.2* | 0.0 |  |  |  | 0.002^**^ | 0.006^*^(hd04T), -0.007^*^(fp99C) | 0.53 | 0.45 |
| *QPFSS.cgb-7A* | *Xwmc488* | 0.0 |  |  |  | -0.015^****^ |  | 2.10 |  |
| *QPFSS.cgb-7B* | *-P3461.1* | 2.6 |  |  |  | 0.009^****^ |  | 0.87 |  |
| ***QPFSS.cgb-3A*** | *Xwmc21* | 0.0 | ***QPFSS.cgb-5B*** | *P4138* | 0.0 | 0.007^****^ |  | 0.72 |  |
| *QPFSS.cgb-2A.1* | *Xwmc382* | 4.0 | *QPFSS.cgb-6B.2* | *-Xgwm132* | 0.9 | 0.004^****^ |  | 0.59 |  |
| *QPFSS.cgb-2A.2* | *P8444.3* | 2.0 | *QPFSS.cgb-6B.2* | *-Xgwm132* | 0.9 | 0.006^****^ |  | 0.22 |  |
| *QPFSS.cgb-4A* | *P3613.2* | 0.0 | *QPFSS.cgb-4B.1* | *Xgwm513* | 0.0 | -0.007^****^ | -0.010^***^(fp99C), 0.012^****^(hd04C) | 0.72 | 0.57 |
| *QPFSS.cgb-4B.1* | *Xgwm513* | 0.0 | ***QPFSS.cgb-5B*** | *P4138* | 0.0 | -0.011^****^ | 0.011^***^(hd04C) | 0.53 | 0.31 |
| *QPFSS.cgb-4B.2* | *Xgwm495* | 4.0 | *QPFSS.cgb-6B.1* | *P3454.2* | 6.0 | -0.009^****^ | -0.008^*^(hd04C) | 0.47 | 0.37 |
| *QPFSS.cgb-4B.2* | *Xgwm495* | 4.0 | ***QPFSS.cgb-5B*** | *P4138* | 0.0 | 0.020^****^ |  | 0.42 |  |
| *QSL.cgb-2B.3* | *-Xwmc223* | 1.6 |  |  |  | -0.106^****^ |  | 1.94 |  |
| ***QSL.cgb-2D.1*** | *Xgwm296.1* | 0.0 |  |  |  | 0.173^****^ |  | 3.09 |  |
| *QSL.cgb-2D.2* | *-Xwmc112* | 2.0 |  |  |  | 0.196^****^ |  | 2.73 |  |
| ***QSL.cgb-2D.3*** | *Xwmc144* | 10.0 |  |  |  | 0.067^****^ |  | 3.47 |  |
| *QSL.cgb-3A* | *P3614* | 0.0 |  |  |  | 0.135^****^ |  | 0.72 |  |
| ***QSL.cgb-3B.1*** | *Xpsp3030* | 0.0 |  |  |  | 0.162^****^ |  | 1.11 |  |
| ***QSL.cgb-3B.2*** | *P3156.1* | 0.0 |  |  |  | 0.177^****^ |  | 2.26 |  |
| *QSL.cgb-4A.1* | *Xwmc89* | 2.0 |  |  |  | -0.225^****^ |  | 2.74 |  |
| *QSL.cgb-4A.2* | *P8222.2* | 0.0 |  |  |  | 0.104^****^ |  | 0.24 |  |
| *QSL.cgb-4B.3* | *Xgwm513* | 0.0 |  |  |  | -0.110^****^ |  | 4.02 |  |
| ***QSL.cgb-4D*** | *Xgwm165.2* | 0.0 |  |  |  | -0.115^****^ |  | 0.56 |  |
| *QSL.cgb-5A.1* | *-Xgwm304* | 1.4 |  |  |  | 0.435^****^ |  | 2.40 |  |
| *QSL.cgb-5A.2* | *P2470* | 0.0 |  |  |  | -0.223^****^ |  | 0.26 |  |
| *QSL.cgb-5A.3* | *-Xgwm443* | 1.7 |  |  |  | -0.212^****^ |  | 0.11 |  |
| ***QSL.cgb-5A.4*** | *P3616.5* | 0.0 |  |  |  | 0.260^****^ |  | 1.26 |  |
| *QSL.cgb-6A* | *-P4232.4* | 1.4 |  |  |  | 0.168^****^ |  | 1.97 |  |
| *QSL.cgb-7A.1* | *-Xgwm635.1* | 4.3 |  |  |  | -0.305^****^ | 0.077^*^(fy01T), 0.078^*^(fy01C) | 6.08 | 0.24 |
| ***QSL.cgb-7A.2*** | *-P3622.1* | 0.5 |  |  |  | -0.183^****^ |  | 2.00 |  |
| ***QSL.cgb-7B*** | *Xwmc273* | 0.0 |  |  |  | -0.126^****^ |  | 1.06 |  |
| ***QSL.cgb-2D.1*** | *Xgwm296.1* | 0.0 | ***QSL.cgb-3B.2*** | *P3156.1* | 0.0 | 0.070^****^ |  | 0.66 |  |
| *QSL.cgb-2D.3* | *Xwmc144* | 10.0 | ***QSL.cgb-3B.1*** | *X3030* | 0.0 | -0.101^****^ |  | 0.57 |  |
| ***QSL.cgb-3B.1*** | *Xpsp3030* | 0.0 | ***QSL.cgb-7B*** | *Xwmc273* | 0.0 | -0.066^****^ |  | 0.00 |  |
| ***QSL.cgb-4D*** | *Xgwm165.2* | 0.0 | ***QSL.cgb-7A.2*** | *-P3622.1* | 0.5 | -0.121^****^ |  | 0.55 |  |
| ***QSL.cgb-5A.4*** | *P3616.5* | 0.0 | ***QSL.cgb-7B*** | *Xwmc273* | 0.0 | 0.100^****^ |  | 0.55 |  |
| *QSL.cgb-1B.1* | *Xgwm131* | 0.0 | *QSL.cgb-5D* | *Xgdm3* | 20.0 | -0.050^***^ |  | 0.34 |  |
| *QSL.cgb-1B.2* | *Xcwm548* | 0.0 | *QSL.cgb-5D* | *Xgdm3* | 20.0 | 0.200^****^ |  | 0.48 |  |
| *QSL.cgb-2B.1* | *P3601* | 6.0 | *QSL.cgb-2D.4* | *Xgwm157* | 4.0 | 0.121^****^ |  | 1.38 |  |
| *QSL.cgb-2B.2* | *-P5322* | 0.3 | *QSL.cgb-2D.5* | *P4233.2* | 2.0 | 0.119^****^ |  | 0.48 |  |
| *QSL.cgb-2B.4* | *Xwmc441* | 0.0 | *QSL.cgb-4B.1* | *Xgwm165.1* | 4.0 | -0.061^****^ |  | 0.23 |  |
| *QSL.cgb-2B.5* | *Xgwm374* | 0.0 | *QSL.cgb-4B.2* | *Xgwm107* | 0.0 | -0.080^****^ |  | 0.23 |  |
| *QDSS.cgb-2D.1* | *Xgwm296.1* | 0.0 |  |  |  | -0.050^****^ |  | 2.79 |  |
| *QDSS.cgb-3A.1* | *P3614* | 0.0 |  |  |  | -0.035^****^ |  | 0.00 |  |
| ***QDSS.cgb-3A.2*** | *-Xwmc21* | 1.5 |  |  |  | 0.057^****^ |  | 1.72 |  |
| ***QDSS.cgb-3B.1*** | *P2076* | 4.0 |  |  |  | -0.051^****^ |  | 4.16 |  |
| ***QDSS.cgb-3B.2*** | *Xcwm539.1* | 0.0 |  |  |  | 0.022^****^ |  | 1.19 |  |
| ***QDSS.cgb-3D*** | *-Xgwm645* | 1.9 |  |  |  | 0.054^****^ |  | 5.42 |  |
| ***QDSS.cgb-5A*** | *P3616.5* | 0.0 |  |  |  | -0.079^****^ |  | 2.28 |  |
| ***QDSS.cgb-5B*** | *Xgwm540* | 0.0 |  |  |  | 0.043^****^ |  | 2.83 |  |
| ***QDSS.cgb-6A.1*** | *Xcwm306* | 4.0 |  |  |  | -0.107^****^ |  | 14.31 |  |
| ***QDSS.cgb-6A.2*** | *Xcwm487* | 4.0 |  |  |  | -0.016^****^ |  | 2.35 |  |
| *QDSS.cgb-6B* | *P6901.3* | 4.0 |  |  |  | 0.083^****^ |  | 6.37 |  |
| *QDSS.cgb-7A.1* | *-Xgwm635.1* | 2.3 |  |  |  | 0.038^****^ |  | 4.41 |  |
| ***QDSS.cgb-7A.2*** | *P6411.1* | 0.0 |  |  |  | 0.046^****^ |  | 2.60 |  |
| ***QDSS.cgb-7B.2*** | *Xgwm68.1* | 4.0 |  |  |  | 0.045^****^ |  | 1.03 |  |
| ***QDSS.cgb-3B.1*** | *P2076* | 4.0 | ***QDSS.cgb-3B.2*** | *Xcwm539.1* | 0.0 | -0.022^****^ |  | 0.19 |  |
| ***QDSS.cgb-3B.2*** | *Xcwm539.1* | 0.0 | ***QDSS.cgb-6A.2*** | *Xcwm487* | 4.0 | -0.020^****^ |  | 0.45 |  |
| ***QDSS.cgb-3D*** | *-Xgwm645* | 1.9 | *QDSS.cgb-4A* | *Xwmc89* | 0.0 | 0.045^****^ |  | 1.82 |  |
| ***QDSS.cgb-3D*** | *-Xgwm645* | 1.9 | ***QDSS.cgb-6A.1*** | *Xcwm306* | 4.0 | -0.048^****^ |  | 0.90 |  |
| ***QDSS.cgb-3D*** | *-Xgwm645* | 1.9 | ***QDSS.cgb-7A.2*** | *P6411.1* | 0.0 | 0.022^****^ |  | 0.82 |  |
| ***QDSS.cgb-5A*** | *P3616.5* | 0.0 | ***QDSS.cgb-5B*** | *Xgwm540* | 0.0 | -0.019^****^ |  | 0.48 |  |
| ***QDSS.cgb-6A.1*** | *Xcwm306* | 4.0 | ***QDSS.cgb-7B.2*** | *Xgwm68.1* | 4.0 | 0.044^****^ |  | 1.46 |  |
| *QDSS.cgb-1D.1* | *Xgdm33* | 2.0 | *QDSS.cgb-2A.2* | *Xwmc401* | 0.0 | -0.015^****^ |  | 0.39 |  |
| *QDSS.cgb-1D.1* | *Xgdm33* | 4.0 | *QDSS.cgb-2A.3* | *Xwmc296* | 0.0 | 0.038^****^ |  | 0.41 |  |
| *QDSS.cgb-1D.2* | *Xwmc432* | 0.0 | *QDSS.cgb-2B.2* | *Xgwm374* | 0.0 | -0.024^****^ |  | 0.02 |  |
| *QDSS.cgb-1D.3* | *Xwmc222* | 0.0 | *QDSS.cgb-2B.1* | *Xwmc474* | 0.0 | 0.023^****^ |  | 0.59 |  |
| *QDSS.cgb-1D.3* | *Xwmc222* | 0.0 | *QDSS.cgb-2A.1* | *Xgwm328* | 0.0 | 0.026^****^ |  | 0.64 |  |
| *QDSS.cgb-2D.2* | *P3470.3* | 0.0 | ***QDSS.cgb-6A.2*** | *Xcwm487* | 4.0 | -0.030^****^ |  | 0.64 |  |
| ***QDSS.cgb-3A.2*** | *-Xwmc21* | 1.5 | *QDSS.cgb-7B.1* | *-P6401* | 7.2 | 0.016^****^ |  | 0.61 |  |
| *QDSS.cgb-3A.3* | *-Xwmc505.2* | 11.4 | *QDSS.cgb-7B.1* | *-P6401* | 7.2 | 0.017^****^ |  | 0.05 |  |
| ***QPH.cgb-1B.1*** | *P3446.1* | 0.0 |  |  |  | 5.4^****^ |  | 4.68 |  |
| ***QPH.cgb-1B.4*** | *P3622.2* | 0.0 |  |  |  | -3.3^****^ |  | 1.93 |  |
| ***QPH.cgb-2A*** | *Xwmc453.2* | 0.0 |  |  |  | -0.5^***^ |  | 0.36 |  |
| ***QPH.cgb-2D.1*** | *Xwmc453.1* | 0.0 |  |  |  | 1.9^****^ |  | 3.47 |  |
| *QPH.cgb-2D.4* | *P3176.1* | 0.0 |  |  |  | 2.3^****^ |  | 1.31 |  |
| ***QPH.cgb-3A*** | *-Xwmc532* | 9.3 |  |  |  | -2.1^****^ |  | 1.23 |  |
| ***QPH.cgb-4A*** | *P6431.1* | 6.0 |  |  |  | 3.1^****^ |  | 0.87 |  |
| ***QPH.cgb-4D*** | *Xgwm192* | 6.0 |  |  |  | 4.3^****^ | -1.5^*^(fp99T), -1.9^***^(fy01T), -1.3^*^(hd05T), 1.9^***^(hd06T) | 5.94 | 0.48 |
| ***QPH.cgb-5A*** | *Xgwm291* | 0.0 |  |  |  | -3.6^****^ |  | 1.42 |  |
| ***QPH.cgb-6B.2*** | *Xgwm132* | 0.0 |  |  |  | 5.1^****^ | -1.3^*^(fp99T), -1.6^***^(fy01T), 1.1^*^(hd06T) | 0.82 | 0.08 |
| ***QPH.cgb-6B.6*** | *Xwmc269.3* | 0.0 |  |  |  | -6.3^****^ | 2.0^***^(fp99T), 2.4^****^(fy01T), -2.0^***^(hd06T), 1.3^*^(fp99C), -1.2^*^(hd05C), -2.0^***^(hd06C) | 4.26 | 0.53 |
| ***QPH.cgb-1B.1*** | *P3446.1* | 0.0 | ***QPH.cgb-4D*** | *Xgwm192* | 6.0 | -2.4^****^ |  | 0.42 |  |
| ***QPH.cgb-2A*** | *Xwmc453.2* | 0.0 | ***QPH.cgb-4A*** | *P6431.1* | 6.0 | 0.9^****^ |  | 0.75 |  |
| ***QPH.cgb-2A*** | *Xwmc453.2* | 0.0 | ***QPH.cgb-6B.6*** | *Xwmc269.3* | 0.0 | -1.3^****^ |  | 0.56 |  |
| ***QPH.cgb-2D.1*** | *Xwmc453.1* | 0.0 | ***QPH.cgb-4A*** | *P6431.1* | 6.0 | -2.4^****^ |  | 1.11 |  |
| ***QPH.cgb-3A*** | *-Xwmc532* | 9.3 | ***QPH.cgb-5A*** | *Xgwm291* | 0.0 | -0.7^***^ |  | 0.40 |  |
| ***QPH.cgb-3A*** | *-Xwmc532* | 9.3 | ***QPH.cgb-6B.6*** | *Xwmc269.3* | 0.0 | 3.9^****^ |  | 0.38 |  |
| ***QPH.cgb-4A*** | *P6431.1* | 6.0 | ***QPH.cgb-5A*** | *Xgwm291* | 0.0 | -1.3^****^ |  | 1.36 |  |
| ***QPH.cgb-6B.2*** | *Xgwm132* | 0.0 | ***QPH.cgb-6B.6*** | *Xwmc269.3* | 0.0 | -1.1^****^ |  | 0.68 |  |
| *QPH.cgb-1A.1* | *P3615.2* | 0.0 | *QPH.cgb-6B.1* | *P3454.1* | 0.0 | -3.1^****^ |  | 0.86 |  |
| *QPH.cgb-1A.2* | *P2478.3* | 0.0 | ***QPH.cgb-1B.4*** | *P3622.2* | 0.0 | -1.6^****^ |  | 0.45 |  |
| *QPH.cgb-1A.3* | *P8222.1* | 0.0 | *QPH.cgb-1B.3* | *P1142.2* | 0.0 | 1.9^****^ |  | 0.12 |  |
| *QPH.cgb-1A.3* | *-Xgwm497.1* | 0.6 | *QPH.cgb-1B.5* | *-P3616.1* | 3.1 | -2.1^****^ |  | 1.69 |  |
| ***QPH.cgb-1B.1*** | *P3446.1* | 0.0 | *QPH.cgb-2D.3* | *-P4233.2* | 8.2 | 0.5^***^ |  | 2.42 |  |
| *QPH.cgb-1B.2* | *P5140.3* | 0.0 | *QPH.cgb-2D.2* | *Xwmc144* | 6.0 | -0.5^**^ |  | 0.23 |  |
| *QPH.cgb-1B.2* | *P5140.3* | 0.0 | *QPH.cgb-7A* | *Xgwm635.1* | 0.0 | -1.6^****^ |  | 0.03 |  |
| *QPH.cgb-1B.2* | *P5140.3* | 0.0 | *QPH.cgb-2D.3* | *-P4233.2* | 5.2 | -3.8^****^ |  | 1.27 |  |
| *QPH.cgb-1B.3* | *P1142.2* | 0.0 | *QPH.cgb-2D.3* | *-P4233.2* | 8.2 | -0.9^****^ |  | 0.00 |  |
| ***QPH.cgb-1B.4*** | *P3622.2* | 0.0 | *QPH.cgb-7A* | *Xgwm635.1* | 0.0 | 2.5^****^ |  | 0.62 |  |
| *QPH.cgb-1D.1* | *Xgdm33* | 0.0 | *QPH.cgb-5D* | *Xgdm68* | 0.0 | -0.5^***^ |  | 0.47 |  |
| *QPH.cgb-1D.2* | *Xcwm1* | 0.0 | *QPH.cgb-5D* | *Xgdm68* | 0.0 | -1.6^****^ |  | 0.15 |  |
| *QPH.cgb-2B* | *-P5322* | 1.3 | *QPH.cgb-6B.3* | *Xcwm29* | 3.0 | -0.7^****^ |  | 0.02 |  |
| *QPH.cgb-2B* | *-P5322* | 1.3 | *QPH.cgb-6B.4* | *T138.1* | 0.0 | -0.4^**^ |  | 0.08 |  |
| *QPH.cgb-3B.1* | *-Xgwm284* | 3.2 | *QPH.cgb-6A* | *Xwmc417.1* | 0.0 | -1.5^****^ |  | 0.89 |  |
| *QPH.cgb-3B.2* | *Xgwm285* | 0.0 | *QPH.cgb-6B.1* | *P3454.1* | 0.0 | -1.8^****^ |  | 0.34 |  |
| *QPH.cgb-3B.2* | *Xgwm285* | 0.0 | *QPH.cgb-6A* | *Xwmc417.1* | 0.0 | -0.6^****^ |  | 0.03 |  |
| *QPH.cgb-3B.3* | *-Xwmc3* | 0.6 | *QPH.cgb-6A* | *Xwmc417.1* | 0.0 | -0.4^***^ |  | 0.09 |  |
| *QPH.cgb-6B.4* | *T138.1* | 0.0 | *QPH.cgb-7B* | *-P1123.2* | 3.7 | -1.7^****^ |  | 0.22 |  |
| *QPH.cgb-6B.5* | *Xgwm193* | 0.0 | *QPH.cgb-7B* | *-P1123.2* | 0.7 | 3.2^****^ |  | 0.74 |  |
| ***QPH.cgb-6B.6*** | *Xwmc269.3* | 0.0 | *QPH.cgb-7B* | *-P1123.2* | 3.7 | 0.9^****^ |  | 0.07 |  |

^1^ YP (g): Yield per plant; NSP: Number of spikes per plant; NGS: Number of grains per spike; TGW (g): 1000-grain weight; TNSS: Total number of spikelets per spike; NSSS: Number of sterile spikelets per spike; PFSS: Proportion of fertile spikelets per spike; SL (cm): Spike length; DSS: Density of spikelets per spike; PH (cm): Plant height

^2^ The bold QTL has both *a* effects and *aa* effects

^3^ marker means the left marker of the marker interval, and“- + marker” indicates the right marker

^4^ dis. is the distance of the related QTL from its nearest marker

^5^ *a*: additive main effects; *aa*: epistatic main effects; *ae*: additive environment interaction effects; *aae*: epistatic environment interaction effects

^6 *^, ^**^, ^***^, ^****^, significant at *P*<0.05, *P*<0.01, *P*<0.005 and *P*<0.0001, respectively

^7^ hd98, hd99, hd00, hd03 and hd04: Haidian, Beijing in 1998, 1999, 2000, 2003 and 2004, respectively; ly99: Luoyang, Henan in 1999; fp99: Fuping, Shaanxi in 1999; fy01: Fenyang, Shanxi in 2001

hd98T: hd98 under drought stressed (DS) treatment; hd98C: hd98 under well-watered (WW) treatment, other similar.
